# Enzyme Complexes of Ptr4CL and PtrHCT Modulate Co-enzyme A Ligation of Hydroxycinnamic Acids for Monolignol Biosynthesis in *Populus trichocarpa*

**DOI:** 10.3389/fpls.2021.727932

**Published:** 2021-10-06

**Authors:** Chien-Yuan Lin, Yi Sun, Jina Song, Hsi-Chuan Chen, Rui Shi, Chenmin Yang, Jie Liu, Sermsawat Tunlaya-Anukit, Baoguang Liu, Philip L. Loziuk, Cranos M. Williams, David C. Muddiman, Ying-Chung Jimmy Lin, Ronald R. Sederoff, Jack P. Wang, Vincent L. Chiang

**Affiliations:** ^1^Forest Biotechnology Group, Department of Forestry and Environmental Resources, North Carolina State University, Raleigh, NC, United States; ^2^Joint BioEnergy Institute, Lawrence Berkeley National Laboratory, Berkeley, CA, United States; ^3^State Key Laboratory of Tree Genetics and Breeding, Northeast Forestry University, Harbin, China; ^4^Department of Electrical and Computer Engineering, North Carolina State University, Raleigh, NC, United States; ^5^Department of Forestry, Beihua University, Jilin, China; ^6^W.M. Keck FTMS Laboratory, Department of Chemistry, North Carolina State University, Raleigh, NC, United States

**Keywords:** protein interaction, monolignol biosynthesis, wood formation, *Populus trichocarpa*, BiFC, metabolic flux

## Abstract

Co-enzyme A (CoA) ligation of hydroxycinnamic acids by 4-coumaric acid:CoA ligase (4CL) is a critical step in the biosynthesis of monolignols. Perturbation of 4CL activity significantly impacts the lignin content of diverse plant species. In *Populus trichocarpa*, two well-studied xylem-specific Ptr4CLs (Ptr4CL3 and Ptr4CL5) catalyze the CoA ligation of 4-coumaric acid to 4-coumaroyl-CoA and caffeic acid to caffeoyl-CoA. Subsequently, two 4-hydroxycinnamoyl-CoA:shikimic acid hydroxycinnamoyl transferases (PtrHCT1 and PtrHCT6) mediate the conversion of 4-coumaroyl-CoA to caffeoyl-CoA. Here, we show that the CoA ligation of 4-coumaric and caffeic acids is modulated by Ptr4CL/PtrHCT protein complexes. Downregulation of *PtrHCTs* reduced Ptr4CL activities in the stem-differentiating xylem (SDX) of transgenic *P. trichocarpa*. The Ptr4CL/PtrHCT interactions were then validated *in vivo* using biomolecular fluorescence complementation (BiFC) and protein pull-down assays in *P. trichocarpa* SDX extracts. Enzyme activity assays using recombinant proteins of Ptr4CL and PtrHCT showed elevated CoA ligation activity for Ptr4CL when supplemented with PtrHCT. Numerical analyses based on an evolutionary computation of the CoA ligation activity estimated the stoichiometry of the protein complex to consist of one Ptr4CL and two PtrHCTs, which was experimentally confirmed by chemical cross-linking using SDX plant protein extracts and recombinant proteins. Based on these results, we propose that Ptr4CL/PtrHCT complexes modulate the metabolic flux of CoA ligation for monolignol biosynthesis during wood formation in *P. trichocarpa*.

## Introduction

Lignin was first described in 1839 as an “incrusting material of wood” by Payen (1839). Since then, there have been many diverse efforts to resolve the function, structure, formation, and biodegradation of this intriguing constituent of the plant secondary cell wall (SCW) (Sarkanen and Ludwig, 1971; Gross, 1979; Higuchi, 1985, 1990; Boerjan et al., 2003; Weng and Chapple, 2010; Ralph et al., 2019). Lignin is one of three major components in the SCW of vascular plants, along with cellulose and hemicelluloses (Sarkar et al., 2009; Albersheim et al., 2010). In the SCW, lignin interweaves with cellulose and hemicelluloses to form lignin–carbohydrate complexes (LCCs) through covalent linkages, such as (phenyl) glycosidic, acetal, ester, and ether bonds (Jung, 1989; Jin et al., 2006; Du et al., 2013, 2014; Tarasov et al., 2018). Depending on the plant species and biomass origin, lignin content can vary from 18–35% in woody plants and 7–30% in herbaceous plants (Sun and Cheng, 2002; Rowell et al., 2005; Pauly and Keegstra, 2008; Gupta et al., 2014).

Lignin is a heterogeneous phenolic polymer and the second most abundant biopolymer on earth, accounting for ∼30% of the organic carbon in the terrestrial biosphere (Boerjan et al., 2003). Lignin is synthesized through oxidative radical coupling (Ralph et al., 2004) and polymerized from three canonical monolignol precursors, 4-coumaryl alcohol, coniferyl alcohol, and sinapyl alcohol, which form the three major subunits of lignin, referred to as 4-hydroxyphenyl (H), guaiacyl (G), and syringyl (S) subunits, respectively (Higuchi, 1997). The composition of lignin is highly malleable (Sederoff et al., 1999; Ralph, 2010; Vanholme et al., 2012; Mottiar et al., 2016) and can vary greatly in the proportion of traditional subunits, in the types of linkages, and in the incorporation of non-canonical monomers into the lignin polymer. The incorporation of dihydroconiferyl alcohol (Ralph et al., 1997), caffeyl alcohol (Chen et al., 2012), ferulate (Wilkerson et al., 2014), tricin (Lan et al., 2015), hydroxystilbene (Carlos del Río et al., 2017), benzoate (Kim et al., 2020), and hydroxycinnamic amides (del Río et al., 2020) in lignin have been documented.

The deposition of lignin is crucial to support the plant body by providing mechanical rigidity, facilitating water transport due to its hydrophobicity, and preventing natural biomass degradation by its highly irregular chemical structure and its resistance to biotic and abiotic stresses (Hu et al., 1999; Tzin and Galili, 2010; Miedes et al., 2014; Bomble et al., 2017). Lignin also confers recalcitrance to lignocellulosic feedstocks for bioenergy and industrial applications, adversely impacting biomass utilization for biofuel and pulp/paper production (Himmel, 2009). Lignin content, composition, and biomass density are significant contributors to biomass recalcitrance (Campbell and Sederoff, 1996; Novaes et al., 2010).

In angiosperms, the lignin is predominantly polymerized from S and G monolignols, while in most gymnosperms, lignin is mainly formed from G monolignols. The relative abundance of H monolignols is usually below 1% in the angiosperms, but it can be higher in monocot grasses (∼5%) (Barrière et al., 2007; Vanholme et al., 2010) or more elevated in compression wood of gymnosperms (Yeh et al., 2006; Nanayakkara et al., 2009). Moreover, within the same plant species, the deposition of different types of monolignols can also vary. For example, in woody dicots, G subunits are enriched in vessels, and S subunits are enriched in fibers (Wang et al., 2018). The spatiotemporal deposition of monolignols within individual cell types could be modulated in an enzyme-specific or cell type-specific manner during monolignol biosynthesis, which remains to be investigated further (Guo et al., 2010; Zhao et al., 2010; Barros and Dixon, 2020).

**FIGURE 1:**
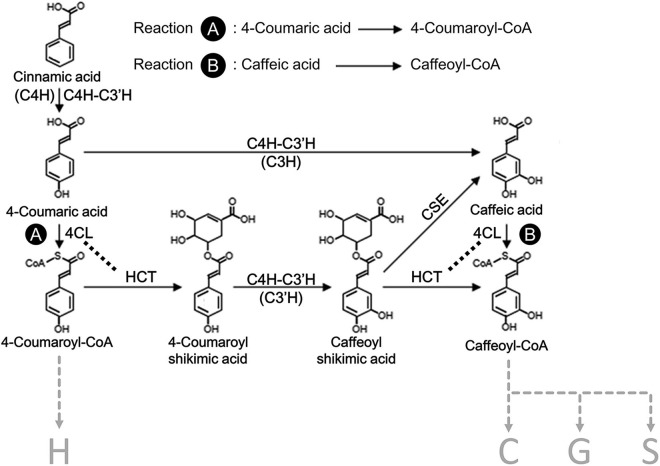
A section of the proposed monolignol biosynthetic pathway in *Populus* spp. The H, C, G, and S refer to the type of subunits in lignin, 4-hydroxyphenyl, catechyl, guaiacyl, and syringyl, respectively. Reaction A indicates 4CL activity for the formation of 4-coumaroyl-CoA from 4-coumaric acid, and reaction B indicates 4CL activity for the formation of caffeoyl-CoA from caffeic acid. C4H, cinnamate 4-hydroxylase; C3H, *p*-coumarate 3-hydroxylase; C3′H, *p*-coumaroyl shikimate 3′-hydroxylase; 4CL, 4-coumarate CoA ligase; HCT, 4-hydroxycinnamoyl-CoA:shikimic acid hydroxycinnamoyl transferase; CSE, caffeoyl shikimate esterase. Enzymes in parenthesis indicate the distinguished reactions for the specific hydroxylation of monolignols.

Monolignol biosynthesis, originates from the shikimate pathway (Brown and Neish, 1955; Vogt, 2010), is often depicted as a biosynthetic grid rather than a linear pathway (Figure 1). The monolignol biosynthetic pathway begins with the conversion of phenylalanine through sequential enzymatic reactions, starting with deamination, followed by hydroxylation, coenzyme A (CoA)-ligation, transesterification, methylation, reduction, and oxidation (Whetten and Sederoff, 1995; Higuchi, 2003; Raes et al., 2003; Hamberger et al., 2007; Liu, 2012; Lu et al., 2013; Shen et al., 2013; Lin et al., 2016; Deng and Lu, 2017). In *Populus trichocarpa*, 23 xylem-specific enzymes within 11 protein families were proposed to be involved in monolignol biosynthesis, which includes ammonia lyase [e.g., phenylalanine ammonia-lyase (PAL)], ligase [e.g., 4-coumaric acid:CoA ligase (4CL)], acyltransferase [e.g., 4-hydroxycinnamoylCoA:shikimic acid hydroxycinnamoyl transferase (HCT)], hydrolase [e.g., caffeoyl shikimate esterase (CSE)], reductase [e.g., cinnamoyl CoA reductase (CCR)], alcohol dehydrogenase [e.g., cinnamyl alcohol dehydrogenase (CAD)], methyltransferase [e.g., caffeoyl-CoA *O*-methyltransferase (CCoAOMT), 5-hydroxyconiferaldehyde *O*-methyltransferase (COMT)], and cytochrome P450 monooxygenases [e.g., cinnamate 4-hydroxylase (C4H), *p*-coumaroyl shikimate 3′-hydroxylase (C3′H), and coniferaldehyde 5-hydroxylase (CAld5H)] (Shi et al., 2010; Saleme et al., 2017; Wang et al., 2018). Recently, in *Brachypodium distachyon* and *Arabidopsis thaliana*, a non-membrane bound cytosolic ascorbate peroxidase that catalyzes the direct 3-hydroxylation of 4-coumarate to caffeate in lignin biosynthesis was identified as a coumarate 3-hydroxylase (C3H), which should be distinguished from the activity of C3′H (Barros et al., 2019). Highly coordinated mechanisms among the monolignol biosynthetic enzymes have been revealed in several model plants and described quantitatively using mathematical modeling (Lee and Voit, 2010; Lee et al., 2012; Wang et al., 2014; Faraji et al., 2015, 2018). Many strategies have been successfully deployed to reduce the recalcitrance of the cell walls to improve biomass utilization by identifying natural variants (MacKay et al., 1997; Studer et al., 2011; Chanoca et al., 2019) or by genetic manipulation of the relative abundance of the monolignol biosynthetic enzymes (Eckardt, 2002; Baucher et al., 2003; Vanholme et al., 2008; Weng et al., 2008; Poovaiah et al., 2014; Peña-Castro et al., 2017; Wang et al., 2018). Conversion of lignin to valued-added bioproducts has also been proposed by lignin valorization (Ragauskas et al., 2014; Upton and Kasko, 2016; Gillet et al., 2017) and lignin manipulation (Welker et al., 2015; Lin and Eudes, 2020).

Although metabolic grids describe pathways as two-dimensional processes, most intracellular enzymes operate as aggregates with tight or loose association within biosynthetic sequences (Nester et al., 1967; Ginsburg and Stadtman, 1970; Winkel, 2009; Sweetlove and Fernie, 2013; Schmitt and An, 2017). These protein aggregates can be multienzyme complexes, which are sometimes conceived to be a set of functionally related and physically associated proteins with a highly organized structure (Ginsburg and Stadtman, 1970). Different protein–protein interactions among the biosynthetic enzymes within the multienzyme complexes can possess physiologically significant regulatory roles, such as compartmentation, metabolite channeling, or catalytic facilitation (Gaertner et al., 1970; Masters, 1977; Stafford, 1981; Luckner, 1984; Hrazdina and Wagner, 1985a; Hrazdina and Jensen, 1992; Winkel, 2004; Williams et al., 2011). Several multienzyme complexes have been described for the biosyntheses of tryptophan (Miles, 2001), shikimate (Giles et al., 1967), polyamines (Panicot et al., 2002), camalexin (Mucha et al., 2019), and cyanogenic glucosides (Laursen et al., 2016). The “supramolecular complexes of sequential metabolic enzymes and cellular structural elements” have been defined as metabolons, which may be anchored on the endoplasmic reticulum (ER) by membrane-bound P450 monooxygenases (P450s) (Srere, 1985; Ovádi and Sreret, 1999; Weid et al., 2004; Jorgensen et al., 2005; Laursen et al., 2015; Obata, 2019).

Since 1974, multienzyme complexes were proposed for aromatic metabolism, including C6-C3 phenolic compounds, flavonoids, and monolignol biosynthesis (Stafford, 1974a, b; Winkel-Shirley, 1999, 2001). The metabolism of flavonoids and monolignols, involving PAL, C4H, and chalcone synthase (CHS), was proposed to be colocalized on the cytoplasmic face of the ER from *Hippeastrum* (Wagner and Hrazdina, 1984), and the involvement of P450s in phenylpropanoid metabolism had been suggested as membrane anchors (Ralston and Yu, 2006). A loose membrane-associated enzyme complex, including PAL, CHS, uridine diphosphate glucose (UDPG):flavonoid glucosyl transferase (UFGT), was revealed in buckwheat (*Fagopyrum esculentum*) to facilitate flavonoid biosynthesis through putative interactions with membrane anchors, such as C4H or flavonoid 3′-hydroxylase (F3′H) (Hrazdina and Wagner, 1985b; Hrazdina et al., 1987; Hrazdina, 1992). Evidence supports a flavonoid metabolon in *A. thaliana* (Burbulis and Winkel-Shirley, 1999), in which CHS and chalcone isomerase (CHI) interact with flavanone 3-hydroxylase (F3H) or dihydroflavonol 4-reductase (DFR) were confirmed by yeast two-hybrid (Y2H) interactions, affinity chromatography (AC) or co-immunoprecipitation (IP) assays (Burbulis and Winkel-Shirley, 1999). The participation of F3′H in the flavonoid metabolon had also been shown in Arabidopsis and rice. Using an Arabidopsis *tt7* mutant that lacks the F3′H, the colocalization of CHS and CHI was disrupted (Winkel-Shirley, 2001; Winkel, 2004), while the association of F3′H with CHS1 has been demonstrated in rice using Y2H (Shih et al., 2008). Differential physical interactions among flavonoid enzymes with flavone synthase II (FNSII) for metabolon formation in flavone/anthocyanin biosynthesis had been elucidated in the plant Order Lamiales (mints, which includes snapdragons and torenia) using split-ubiquitin Y2H and biomolecular fluorescence complementation (BiFC) (Fujino et al., 2018). An isoflavonoid metabolon was also confirmed in soybean with the involvement of soluble enzymes [CHS, CHI, chalcone reductase (CHR)], and twin membrane-bound anchors [C4H and isoflavones synthase (IFS)] (Dastmalchi et al., 2016; Waki et al., 2016; Mameda et al., 2018). A bottleneck for isoflavone production was previously identified by the competition for flavanone between IFS and flavonoid biosynthesis (Liu et al., 2002).

In contrast to the more extensive understanding of multienzyme complexes involved in flavonoid biosynthesis, the identification of protein-protein interaction among monolignol biosynthetic enzymes has been long-postulated but not unambiguously established (Wagner and Hrazdina, 1984; Hrazdina and Wagner, 1985b; Winkel-Shirley, 1999; Biała and Jasiński, 2018). A membrane-bound enzyme complex was first reported at the entry point of the phenylpropanoid pathway using microsomal membrane fractions from potato tubers (Czichi and Kindl, 1975). The PAL-C4H association can use cinnamic acid formed by the PAL reaction as a more effective substrate than cinnamic acid added exogenously (Czichi and Kindl, 1977), while the disruption of the PAL-C4H interaction during the preparation of microsomes led to increased conversion of exogenous cinnamic acid using buckwheat seedlings (Hrazdina and Wagner, 1985b). Direct interactions for PAL-C4H were demonstrated in *Nicotiana tabacum*, by *in vivo* isotopic labeling and fluorescence energy resonance transfer (FRET) microscopy, to strengthen the evidence for metabolic channeling for the entry point of monolignol biosynthesis (Rasmussen and Dixon, 1999; Achnine et al., 2004).

Besides the PAL-C4H interaction, protein-protein interactions among the monolignol biosynthetic enzymes have been discovered around the early steps of the phenylpropanoid pathway through the advancements in plant biotechnology. In Arabidopsis, CYP73A5 (AtC4H) and CYP98A3 (AtC3′H) are colocalized on the ER membrane where they form homo- and heteromers, which is confirmed by fluorescence microscopy and tandem affinity purification (TAP) (Bassard et al., 2012). Two membrane steroid-binding proteins (AtMSBP1 and AtMSBP2) were recently identified to function as a scaffold to interact with all of the three monolignol P450 enzymes (AtC3′H, AtC4H, and AtF5H) by Y2H, BiFC, LC-MS, and IP assays (Gou et al., 2018). In *Populus trichocarpa*, a multienzyme membrane-bound protein complex among monolignol biosynthetic enzymes of PtrC3′H3/PtrC4H1/PtrC4H2 has been identified to catalyze both 4- and 3-hydroxylation of cinnamic acid derivatives in monolignol biosynthesis, which drastically increased enzyme metabolic efficiency (Chen et al., 2011). A heterotetrameric protein complex of Ptr4CL3-Ptr4CL5 was identified with novel enzymatic specificity, and the metabolic regulation of CoA ligation by the complex has been quantitatively described by predictive mathematical modeling (Chen et al., 2014).

Following the proposed metabolic sequence for the early steps of monolignol biosynthesis, HCT is expected to be the downstream partner of 4CL (Figure 1). Based on advanced fluorescence microscopy, a closer association of the ER membrane with At4CL1 and AtHCT was proposed for Arabidopsis (Bassard et al., 2012). However, the physiological function of the protein-protein interaction between 4CL and HCT has not been further characterized. To extend the understanding of the proposed lignin metabolon, we investigated the protein-protein interaction between Ptr4CL and PtrHCT in *P. trichocarpa* with a focus on the CoA-ligation function of Ptr4CL, which is crucial for the regulation of phenylpropanoid metabolic flux. First, we generated PtrHCT downregulated transgenic *P. trichocarpa* using RNA interference (RNAi). When PtrHCT1 or PtrHCT6 was downregulated in the PtrHCT RNAi transgenic plants, we observed a PtrHCT-dependent reduction in the CoA-ligation activities of Ptr4CLs, indicating a Ptr4CL-PtrHCT interaction. Second, we confirmed the Ptr4CL-PtrHCT protein-protein interaction using BiFC and pull-down assays. The Ptr4CL-PtrHCT enzyme complex was confirmed *in vitro* using purified recombinant enzymes. Using enzyme assays of mixed recombinant proteins, a numerical model was constructed to describe the behavior of Ptr4CLs in the Ptr4CL-PtrHCT complex and to predict the stoichiometry of the protein complex based on evolutionary computation (Chen et al., 2014). The mathematical model estimated the stoichiometry of the Ptr4CL-PtrHCT complex to be one subunit of Ptr4CL and two subunits of PtrHCT. Finally, the Ptr4CL-PtrHCT enzyme complex was validated by chemical cross-linking in wood forming tissues of *P. trichocarpa*, supporting the stoichiometry and molecular weight of the predicted Ptr4CL-PtrHCT complex.

## Results

### Co-enzyme A Ligation Activity of Ptr4CL Is Reduced When PtrHCT Is Downregulated

The CoA ligation of hydroxycinnamic acids by 4CL creates an activated state of the acids in phenylpropanoid metabolism for monolignol biosynthesis. To investigate whether HCT interacts with 4CL and the biological significance of an interaction, we measured the 4CL activity when the expression of HCT was downregulated by RNA interference (RNAi). Previously, in *P. trichocarpa*, PtrHCT1 and PtrHCT6 were identified as two xylem-specific and xylem-abundant members of the PtrHCT family involved in monolignol biosynthesis (Shi et al., 2010). Three independent transgenic lines with specific downregulation of PtrHCT1 or PtrHCT6 by RNAi transgenesis were obtained (Supplementary Figure 1), which were used to evaluate how perturbations of PtrHCT may affect the activity, transcript level, and protein abundance of Ptr4CL.

**FIGURE 2:**
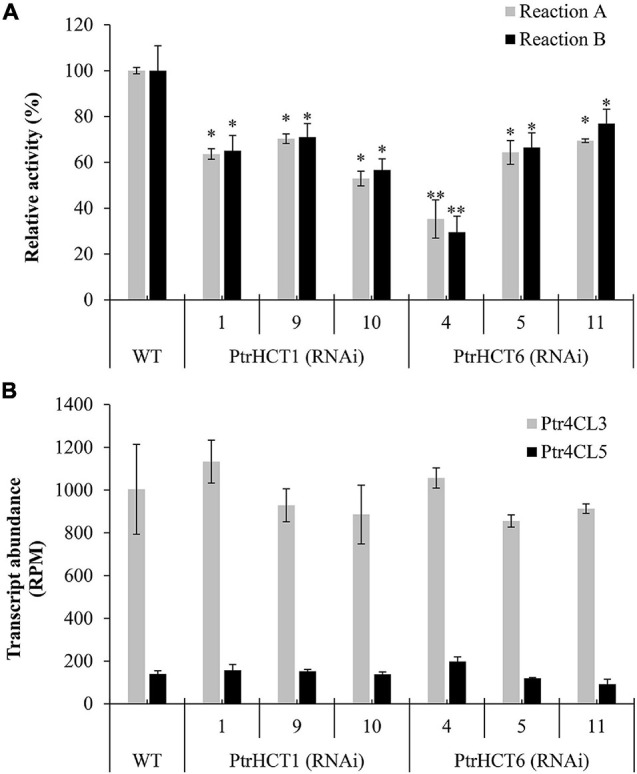
Transcript abundance of SDX-specific Ptr4CLs and their activities in PtrHCT1 or PtrHCT6 RNAi-downregulated transgenic lines. **(A)** The CoA ligation activities toward 4-coumaric acid (Reaction A) and caffeic acid (Reaction B) using SDX extracts of WT, PtrHCT1, and PtrHCT6 RNAi-downregulated transgenic lines. The activities of Ptr4CL were measured using SDX extracts at 37°C for 30 min with 50 μM substrate (final concentration) in the assay solution [50 mM Tris–HCl buffer (pH 7.5), 2 mM MgCl_2_, 2 mM ATP, and 0.2 mM CoA]. **(B)** Transcript abundance of Ptr4CL3 (in gray) and Ptr4CL5 (in black) in WT, PtrHCT1, and PtrHCT6 RNAi-downregulated transgenic lines. RPM, reads per million. Error bars represent the mean ± standard error (SE) from three biological replicates (*n* = 3). Statistical testing was performed using Student’s *t*-test (^∗^*p* < 0.05; ^∗∗^*p* < 0.01).

First, crude protein extracts of stem-differentiating xylem (SDX) were used to measure the CoA ligation activities of 4CL toward 4-coumaric acid (**Reaction A**) and caffeic acid (**Reaction B**). Compared to wildtype (WT), 4CL activity was reduced by 30–47% in reaction A and by 29–43% in reaction B when PtrHCT1 was downregulated; while in the case of PtrHCT6 downregulation, the 4CL activity was also reduced by 31-65% in reaction A and by 23-70% in reaction B (Figure 2A). We then confirmed that the reduction in 4CL activity was not a result of reduced 4CL expression. Full transcriptome RNA-seq showed no significant difference in the transcript abundance of Ptr4CL3 or Ptr4CL5 between the HCT transgenics and WT *P. trichocarpa* (Figure 2B) and the absolute protein abundances of Ptr4CL3 and Ptr4CL5 were also consistent in the SDX of HCT transgenics and WT using protein cleavage isotope dilution mass spectrometry (PC-IDMS) (Supplementary Figure 2A). The specific downregulation of either PtrHCT1 or PtrHCT6 in the corresponding transgenic lines (Supplementary Figure 2B) suggests that the catalytic facilitation of Ptr4CL reactions by PtrHCT is specific, therefore PtrHCT may play a regulatory role in the CoA-ligation activities.

### Protein–Protein Interactions Between Ptr4CL and PtrHCT Revealed by BiFC

The presence of protein–protein interactions between Ptr4CLs and PtrHCTs was investigated using reciprocal BiFC. By fusing the Ptr4CLs or PtrHCTs to the N-terminal fragment of YFP (YFP^N^) or the C-terminal fragment of YFP (YFP^C^), we constructed eight BiFC vectors (Ptr4CL3-YFP^N^, Ptr4CL3-YFP^C^, Ptr4CL5-YFP^N^, Ptr4CL5-YFP^C^, PtrHCT1-YFP^N^, PtrHCT1-YFP^C^, PtrHCT6-YFP^N^, and PtrHCT6-YFP^C^) to evaluate their interactions systematically. Pair-wise combinations of BiFC vectors were co-transfected into *P. trichocarpa* SDX protoplasts. Positive YFP fluorescence was recorded as indication of Ptr4CL and PtrHCT interactions.

**FIGURE 3:**
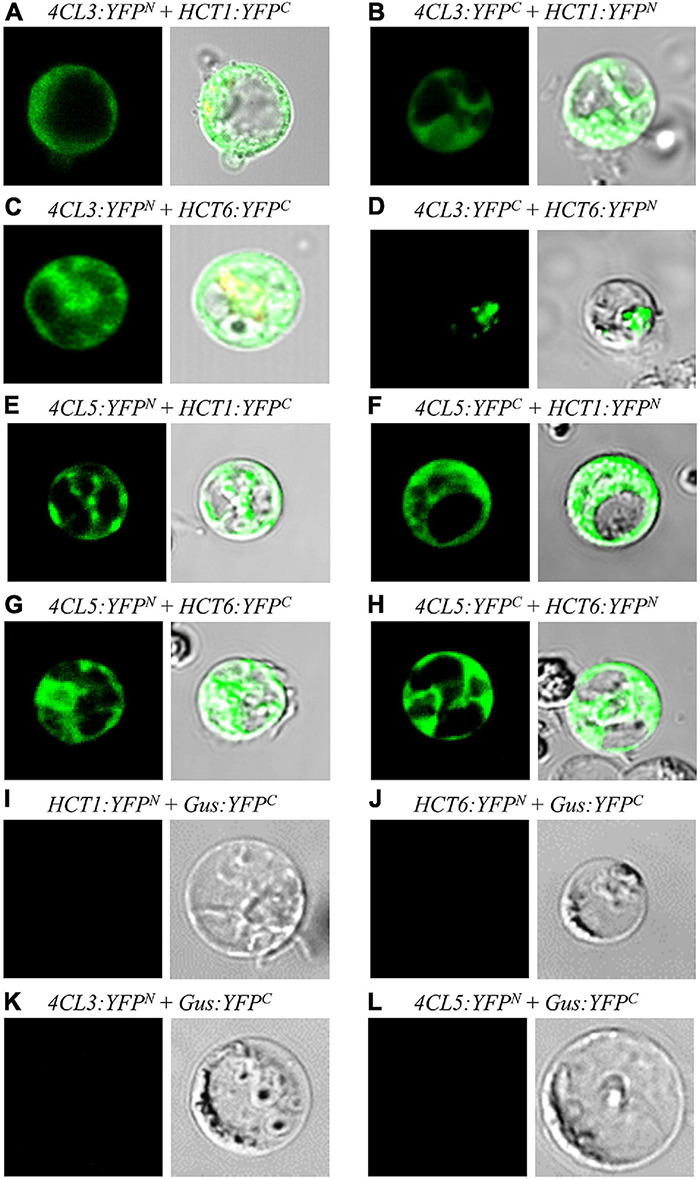
Protein-protein interactions between Ptr4CLs and PtrHCTs were detected by reciprocal bimolecular fluorescence complementation (BiFC). **(A)** Ptr4CL3-YFP^N^ was cotransformed with PtrHCT1-YFP^C^. **(C)** Ptr4CL3-YFP^N^ was cotransformed with PtrHCT6-YFP^C^. **(E)** Ptr4CL5-YFP^N^ was cotransformed with PtrHCT1-YFP^C^. **(G)** Ptr4CL5-YFP^N^ was cotransformed with PtrHCT6-YFP^C^. (**B,D,F,H**) Reciprocal BiFC assays of **(A,C,E,G)**. **(I–L)** Negative controls of BiFC assays. The Gus-YFP^C^ were cotransformed with PtrHCT1-YFP^N^, PtrHCT6-YFP^N^ **(E)**, or Ptr4CL3-YFP^N^, Ptr4CL5-YFP^N^ **(F)**, as the negative controls.

Bright YFP fluorescence signals were detected in *P. trichocarpa* SDX protoplasts when Ptr4CL3-YFP^N^ was co-transfected with either PtrHCT1-YFP^C^ or PtrHCT6-YFP^C^ (Figures 3A,C) and, as well as, when Ptr4CL5-YFP^N^ was co-transfected with either PtrHCT1-YFP^C^ or PtrHCT6-YFP^C^ (Figures 3E,G). Furthermore, the fluorescence signals of YFP were observed for reciprocal pair-wise combinations (Figures 3B,D,F,H). A negative control was included to validate the results of the BiFC assays by co-transfecting a β-glucuronidase (Gus)-fused YFP fragment individually with the eight BiFC vectors. No fluorescence signal was detected when Gus-YFP^C^ was co-transfected with Ptr4CL3-YFP^N^, Ptr4CL5-YFP^N^, PtrHCT1-YFP^N^, and PtrHCT6-YFP^N^ (Figures 3I–L), confirming the specificity of the interactions. The BiFC results revealed that Ptr4CLs (Ptr4CL3 and Ptr4CL5) might directly or indirectly interact with PtrHCTs (PtrHCT1 and PtrHCT6) in *P. trichocarpa*.

### Protein Pull-Down Assays Support the Formation of Ptr4CL-PtrHCT Complexes

**FIGURE 4:**
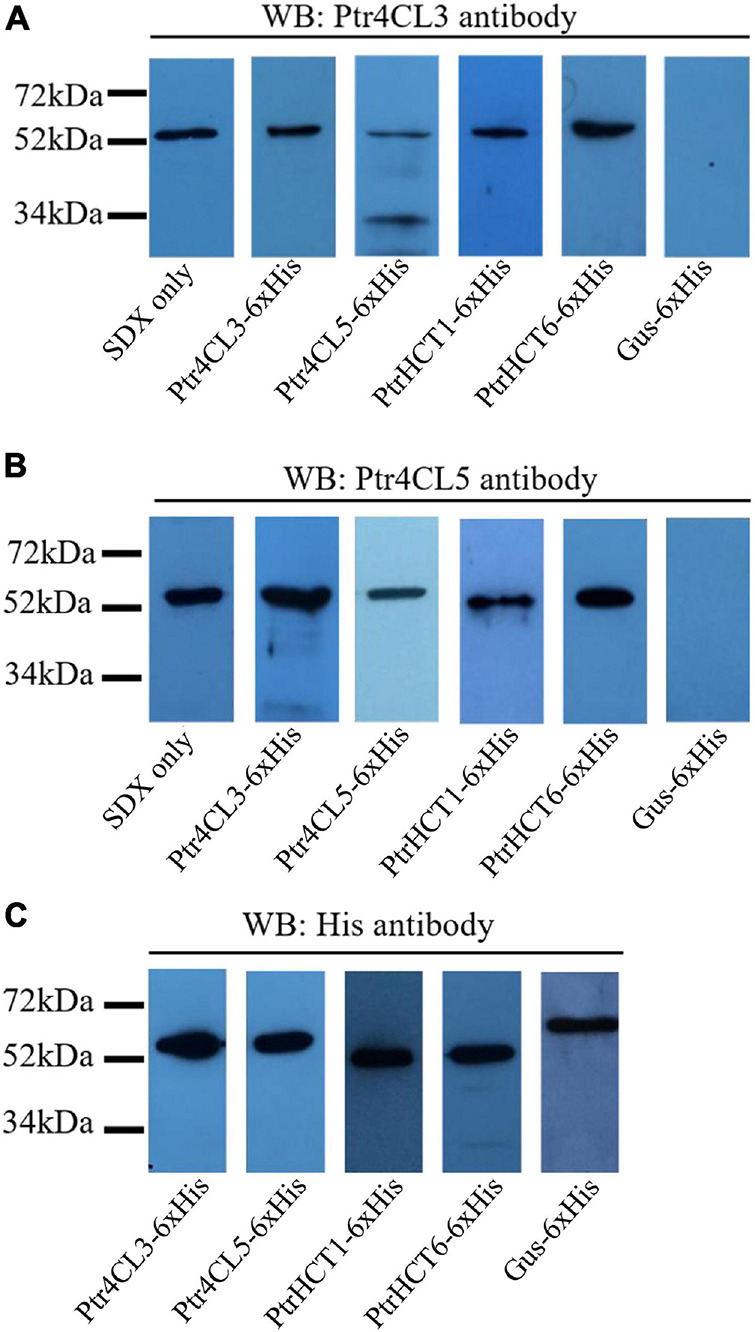
Protein–protein interactions between Ptr4CLs and PtrHCTs were detected on western blots after pull-downs using SDX total protein extracts. SDX total protein extracts were independently incubated with recombinant proteins of Ptr4CL3, Ptr4CL5, PtrHCT1, and PtrHCT6 with a C-terminal 6 × His tag. The protein mixtures were affinity purified and analyzed by western blotting using anti-Ptr4CL3 **(A)** and anti-Ptr4CL5 **(B)** antibodies to identify interacting proteins. Recombinant proteins of Ptr4CL3, Ptr4CL5, PtrHCT1, PtrHCT6, and Gus were detected using His antibody **(C)**. WB, western blot. Refer to Supplementary Figure 6 for the uncropped western blots.

We performed pull-down assays in SDX protein extracts of *P. trichocarpa* to verify the interactions between Ptr4CLs and PtrHCTs (Figure 4). Polyclonal antibodies for Ptr4CLs and PtrHCTs were produced and validated for their protein specificity in SDX extracts and purified recombinant proteins using western blotting. All four antibodies (anti-Ptr4CL3, anti-Ptr4CL5, anti-PtrHCT1, and anti-PtrHCT6) could specifically bind to their target proteins with minimal non-specific binding to other members of their protein families, confirming the suitability of these antibodies for use in pull-down assays (Supplementary Figure 3).

If stable interactions exist between PtrHCTs and Ptr4CLs, the protein complexes could be separated from SDX crude extracts by affinity purification of one of the interacting proteins. The other interacting proteins could then be identified by western blotting using the protein-specific antibodies. Recombinant proteins of Ptr4CL3, Ptr4CL5, PtrHCT1, and PtrHCT6 fused with C- terminal six-histidine-tags (6 × His) were individually mixed with SDX crude extracts and then purified by immobilized nickel-affinity chromatography to isolate their interacting proteins. Gus-6 × His mixed with SDX crude extracts was included as a negative control. When Ptr4CL3-6 × His, Ptr4CL5-6 × His, PtrHCT1-6 × His, and PtrHCT6-6 × His were affinity purified from SDX, Ptr4CL3 was detected in all the pull-down samples using protein-specific antibodies (Figure 4A). Consistently, Ptr4CL5 was also detected as an interacting protein in all the pull-down samples (Figure 4B). None of the target proteins were detected when Gus-6 × His was pulled down from SDX crude extracts, confirming the specificity of the assays for identifying interacting proteins (Figures 4A,B). As positive controls, all the His-tag-fused recombinant proteins were able to be detected using His-tag antibody (Figure 4C). The results of the pull-down assays provided further evidence that PtrHCT interacts with Ptr4CL to form protein complexes *in vivo*.

### Catalytic Activation of CoA-Ligation Activity in Ptr4CLs When Supplemented With PtrHCTs *in vitro*

To investigate the role of PtrHCTs and their effects on CoA-ligation in a Ptr4CL-PtrHCT complex, we used recombinant proteins to study changes in the Ptr4CL activity when PtrHCT is supplemented in the reaction mixture. CoA ligation activity with 4-coumaric acid (**Reaction A**) and caffeic acid (**Reaction B**) as substrates were measured for mixed enzyme assays of Ptr4CL (10 nM) and PtrHCT (40 nM). As a control, Ptr4CL reactions were supplemented with 40 nM of bovine serum albumin (BSA).

**FIGURE 5:**
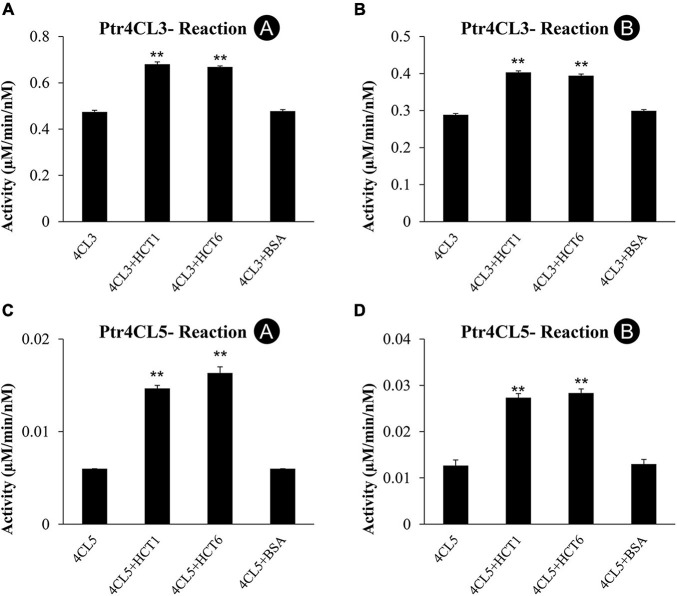
CoA ligation activity with 4-coumaric acid (Reaction A) and caffeic acid (Reaction B) of recombinant Ptr4CL3 and Ptr4CL5 are affected by the presence of PtrHCT1 or PtrHCT6. **(A)** Reaction A of Ptr4CL3 with PtrHCTs. **(B)** Reaction B of Ptr4CL3 with PtrHCTs. **(C)** Reaction A of Ptr4CL5 with PtrHCTs. **(D)** Reaction B of Ptr4CL5 with PtrHCTs. Ptr4CLs were fixed at 10 nM, and PtrHCT concentration was fixed at 40 nM. Substrate concentration was fixed at 50 μM. Error bars represent one SE of three technical replicates. Statistical testing was performed using the Student’s *t*-test (**p < 0.01).

Ptr4CL3 activity was measured when supplemented with PtrHCT1 or PtrHCT6. Compared to Ptr4CL3 alone, the Ptr4CL3 activity toward 4-coumaric acid (**Reaction A**) increased by 43.4% when PtrHCT1 was added and by 40.9% when PtrHCT6 was added (Figure 5A). For caffeic acid (**Reaction B**), Ptr4CL3 activity increased by 39.7% and 36.6% when PtrHCT1 and PtrHCT6 were supplemented, respectively (Figure 5B). For **Reaction A** and **Reaction B**, the Ptr4CL3 activities did not significantly change when BSA was added (Figures 5A,B). Similar to Ptr4CL3, the activity of Ptr4CL5 also increased when PtrHCT1 or PtrHCT6 was present. For **Reaction A**, the activity of Ptr4CL5 increased by 164.7% when PtrHCT1 was present and by 140.5% when PtrHCT6 was present (Figure 5C). For **Reaction B**, Ptr4CL5 activity increased by 124.2% when PtrHCT1 was present and by 113.8% when PtrHCT6 was present (Figure 5D). The addition of BSA to the Ptr4CL5 activity assays also did not alter the CoA ligation rate (Figures 5A–D).

The catalytic activations of Ptr4CLs by PtrHCTs in the *in vitro* enzyme assays are consistent with the findings that Ptr4CL activity is reduced when the expression of PtrHCTs was suppressed in the PtrHCT RNAi transgenic lines *in vivo*. These results indicate a regulatory role of the PtrHCT for Ptr4CL activities in the Ptr4CL-PtrHCT protein complex. However, the enhancement of Ptr4CL activities by PtrHCTs might be underestimated due to the extent of Ptr4CL-PtrHCT complex formation in the *in vitro* enzyme assays.

### Non-additive Co-enzyme A-Ligation Activity Due to PtrHCT Indicates a Specific Interaction Between Ptr4CL and PtrHCT

Changes in enzyme activities mediated by the interactions between Ptr4CL and PtrHCT can be used to infer the stoichiometry of the protein complex. We investigated how Ptr4CL activities would affected the conversion of 4-coumaric acid (**Reaction A**) or caffeic acid (**Reaction B**) when the molar concentration of PtrHCT was varied (from 0 to 40 nM), while the molar concentration of Ptr4CL was fixed at 10 nM.

**FIGURE 6:**
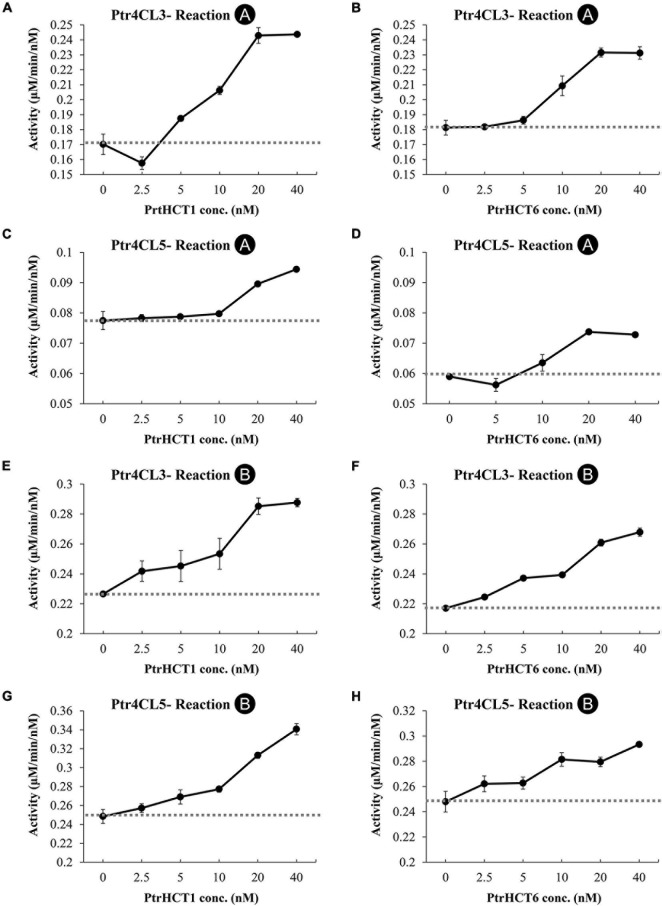
Impact of Ptr4CL-PtrHCT complex formation on the CoA ligation of 4-coumaric acid (Reaction A) or caffeic acid (Reaction B). **(A)** Reaction A of Ptr4CL3 with increasing PtrHCT1 concentration. **(B)** Reaction A of Ptr4CL3 with increasing PtrHCT1 concentration. **(C)** Reaction A of Ptr4CL5 with increasing PtrHCT1 concentration. **(D)** Reaction A of Ptr4CL5 with increasing PtrHCT6 concentration. **(E)** Reaction B of Ptr4CL3 with increasing PtrHCT1 concentration. **(F)** Reaction B of Ptr4CL3 with increasing PtrHCT1 concentration. **(G)** Reaction B of Ptr4CL5 with increasing PtrHCT1 concentration. **(H)** Reaction B of Ptr4CL5 with increasing PtrHCT6 concentration. The concentration of Ptr4CL3 or Ptr4CL5 was fixed as 10 nM. Dashed lines indicate the baseline of activity of each Ptr4CL. Error bars represent SE of three replicates.

The CoA ligation activity when only Ptr4CL3 or Ptr4CL5 is present was taken as the baseline rate (dashed lines in Figure 6). The activities of Ptr4CL3 and Ptr4CL5 toward 4-coumaric acid (**Reaction A**) are shown in Figures 6A–D. The activities of both enzymes increased when the molar concentration of PtrHCTs became higher. The increasing activity plateaued when the Ptr4CL-PtrHCT ratio is 1:2, except for Ptr4CL5-PtrHCT1, yielding the highest activity with a ratio of 1:4 (Figure 6C). The activities of Ptr4CL3 and Ptr4CL5 toward caffeic acid (**Reaction B**) were also examined and shown in Figures 6E–H. Similar to the case of **Reaction A**, both the activities of Ptr4CL3 and Ptr4CL5 for Reaction B were increased when the molar concentration of PtrHCTs became higher. An elevated CoA ligation activity was observed when the ratio of Ptr4CL and PtrHCT was around 1:2, except for Ptr4CL5 with PtrHCT6 that showed the elevated CoA ligation activity at 1:1 ratio (Figure 6H). However, the plateau effect for caffeic acid is less evident than in the 4-coumaric acid reaction for both Ptr4CL3 and Ptr4CL5. Taken together, non-linear deviations from the Ptr4CL activity baseline when reactions were supplemented with PtrHCT were consistently observed (Figure 6). The non-additive increase in the CoA-ligation activities when Ptr4CL interacts with PtrHCT indicated a specific formation of the complex. These quantitative data were then used for mechanistic modeling and numerical analysis to investigate the stoichiometry of the Ptr4CL-PtrHCT complex.

### Mechanistic Modeling and Stoichiometry of Ptr4CL-PtrHCT Protein Complexes

To explore the stoichiometry of the Ptr4CL-PtrHCT complex, a quantitative model was developed to describe how the Ptr4CL-PtrHCT complex affects the overall reaction rate of Ptr4CL. The model was developed by a rule-based algorithm and an evolutionary computational optimization approach based on experimental estimates of enzyme activity (Supplementary Figure 4). Rule-based modeling can represent complex biochemical networks by building an interaction topology model between enzymes and substrates. The evolutionary computation method suggests the most suitable model through automatically mixing and matching potential interaction forms. This model contains both mechanistic modeling and numerical analysis, which has been successfully applied to reveal the functional redundancy of PtrCAld5Hs and the heterotetrameric interactions of the Ptr4CL complex (Wang et al., 2012; Chen et al., 2014; Song, 2014).

The model is based on experimental data and the mixed enzyme activity of Ptr4CLs in the Ptr4CL-PtrHCT complex (Figures 5, 6). The CoA ligation activities were given a fixed concentration of Ptr4CLs with different ratios of PtrHCTs toward 4-coumaric acid or caffeic acid were evaluated by numerical analysis according to the parameters for enzyme activity and enzyme ratios (Figure 6). The numerical analyses were used to measure the overall accuracy for stoichiometry predictions of the Ptr4CL-PtrHCT complex, evaluating the goodness-of-fit of models to the experimental data (Supplementary Figure 5). The model incorporated the enzyme activity under different ratios of Ptr4CL and PtrHCT and can indicate the stoichiometry of the complex based on the root mean square error (RMSE) and the Bayesian information criterium (BIC) (Schwarz, 1978). The parameters for optimizing the mechanistic modeling and the prediction of the stoichiometry of the Ptr4CL-PtrHCT complex were listed in Supplementary Table 2 toward 4-coumaric acid (**Reaction A**) and Supplementary Table 3 toward caffeic acid (**Reaction B**).

**FIGURE 7:**
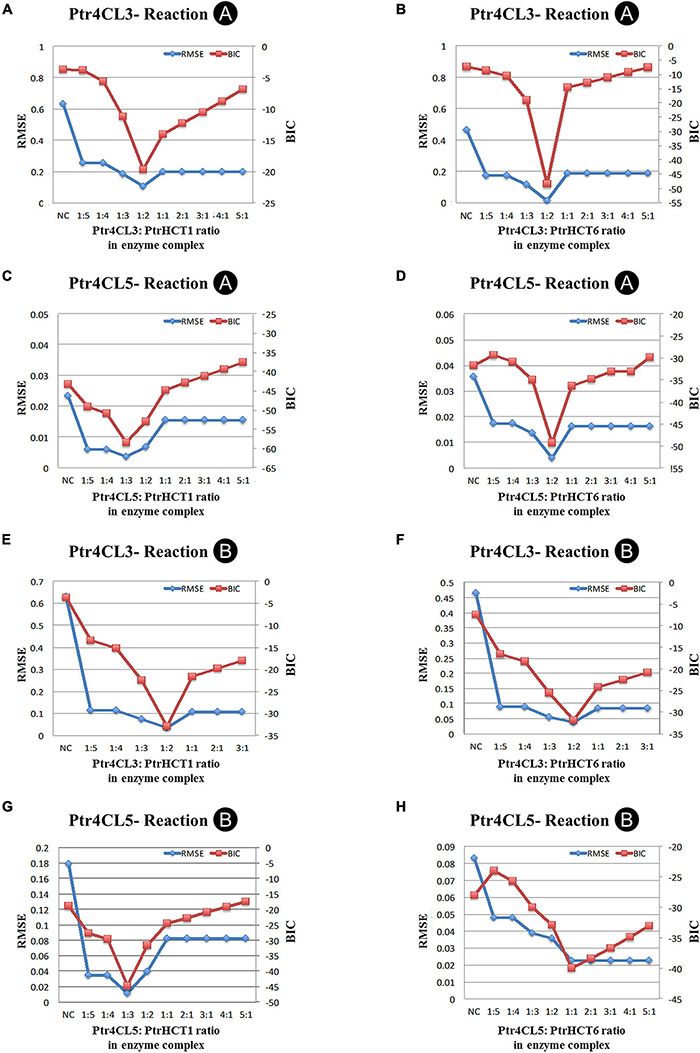
Numerical analysis of CoA ligation activity for 4-coumaric acid (Reaction A) or caffeic acid (Reaction B) in model optimization process. **(A)** Ptr4CL3 to PtrHCT1 ratio for Reaction A. **(B)** Ptr4CL3 to PtrHCT6 ratio for Reaction A. **(C)** Ptr4CL5 to PtrHCT1 ratio for Reaction A. **(D)** Ptr4CL5 to PtrHCT6 ratio for Reaction A. **(E)** Ptr4CL3 to PtrHCT1 ratio for Reaction B. **(F)** Ptr4CL3 to PtrHCT6 ratio for Reaction B. **(G)** Ptr4CL5 to PtrHCT1 ratio for Reaction B. **(H)** Ptr4CL5 to PtrHCT6 ratio for Reaction B. RMSE, root mean squared error; BIC, Bayesian information criterion.

In most cases, both RMSE and BIC had the lowest values when the Ptr4CL to PtrHCT ratio is 1 to 2 (Figures 7A,B,D,E,F), except Ptr4CL5 showed a 1 to 3 ratio preference with PtrHCT1 for **Reaction A** and **Reaction B** (Figures 7C,G) and a 1 to 1 ratio preference with PtrHCT6 for **Reaction B** (Figure 7H). Although the numerical analysis indicates that the most likely protein complex is a heterotrimer of Ptr4CL-PtrHCT with one subunit of Ptr4CL and two subunits of PtrHCTs, other possible combinations predicted for the Ptr4CL5-PtrHCTs enzyme complex indicate there may be different specific interactions between Ptr4CLs and PtrHCTs when different isoforms become the predominant partners during monolingnol biosynthesis.

### The Ptr4CL-PtrHCT Complex Formation *in vivo* in SDX

**FIGURE 8:**
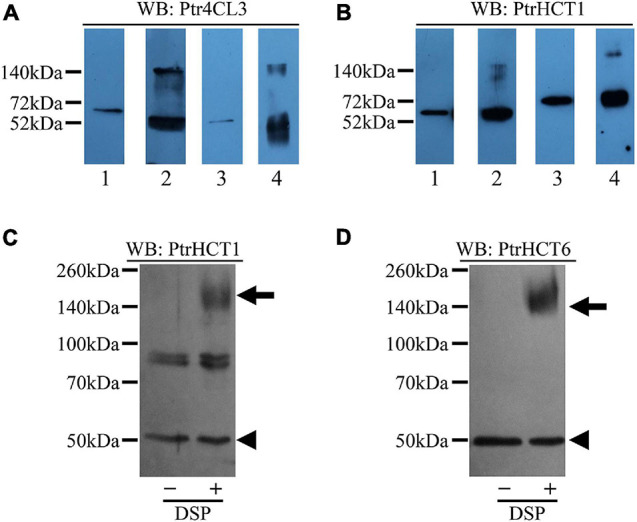
Detection of Ptr4CL-PtrHCT complexes in SDX extracts and recombinant proteins using chemical cross-linking by DSP. **(A)** Cross-linking of recombinant protein Ptr4CLs and PtrHCTs and detection by Ptr4CL3 specific antibody. Lane 1: Non-cross-linked recombinant Ptr4CL3 and PtrHCT1. Lane 2: Cross-linked recombinant Ptr4CL3 and PtrHCT1. Lane 3: Non-cross-linked recombinant Ptr4CL3 and PtrHCT6. Lane 4: Cross-linked recombinant Ptr4CL3 and PtrHCT6. **(B)** Cross-linking of recombinant protein Ptr4CLs and PtrHCTs and detection by PtrHCT1 antibody. Lane 1: Non-cross-linked recombinant Ptr4CL3 and PtrHCT1. Lane 2: Cross-linked recombinant Ptr4CL3 and PtrHCT1. Lane 3: Non-cross-linked recombinant Ptr4CL5 and PtrHCT1. Lane 4: Cross-linked recombinant Ptr4CL5 and PtrHCT1. **(C)** Detection of Ptr4CL-PtrHCT complexes in DSP cross-linked SDX extracts using PtrHCT1 specific antibody. **(D)** Detection of Ptr4CL-PtrHCT complex in DSP cross-linked SDX extract using PtrHCT6 specific antibody. WB, western blot. Refer to Supplementary Figure 6 for the uncropped western blots.

To obtain physical evidence for the stoichiometry of the Ptr4CL-PtrHCT complex, we mixed recombinant proteins of Ptr4CL and PtrHCT with a chemical cross-linker, dithiobis [succinimidyl propionate] (DSP) (Lomant and Fairbanks, 1976). The cross-linking of PtrHCT and Ptr4CL recombinant proteins was carried out with equal concentration (200 nM) of the interacting proteins. The cross-linked proteins were then resolved by SDS-PAGE, and detected using protein-specific antibodies. Using an anti-Ptr4CL3 antibody, Ptr4CL3 without cross-linking showed a protein band of ∼60 kDa, consistent with the molecular weight of a monomeric unit of Ptr4CL3 (Figure 8A, Lines 1 and 3). Cross-linking of Ptr4CL3 with PtrHCT1 or PtrHCT6 (Figure 8A, Lines 2 and 4) revealed a much larger protein band (∼160 kDa) in addition to the monomer, suggesting the presence of a protein complex. Similarly, using PtrHCT1 specific antibody for western blot detection, a protein band (∼160 kDa) was detected after cross-linking PtrHCT1 with Ptr4CL3 or Ptr4CL5 (Figure 8B, Lines 2 and 4). The protein band (∼160 kDa) approximates the molecular weight of the Ptr4CL-PtrHCT complex at a 1:2 ratio predicted by the numerical analysis.

Furthermore, to obtain physiological evidence for the Ptr4CL-PtrHCT complex *in vivo*, we investigated the existence of the Ptr4CL-PtrHCT complex in crude SDX extracts of *P. trichocarpa*. The SDX extracts were cross-linked using DSP, resolved by SDS-PAGE, and detected using anti-PtrHCT antibodies. Compared to SDX extracts without chemical cross-linking, a protein band of ∼160 kDa was detected using PtrHCT1 specific antibody (Figure 8C), as well as PtrHCT6 specific antibody (Figure 8D). The estimated size of the bands is consistent with the expected size of the heterotrimeric Ptr4CL-PtrHCT complex for one unit of Ptr4CL and two units of PtrHCT. These results confirmed the presence of a Ptr4CL-PtrHCT complex when both proteins are present in the recombinant protein mixtures *in vitro* and in the SDX *in vivo*. The stoichiometry of the Ptr4CL-PtrHCT complex is likely to be a 1 to 2 ratio.

## Discussion

In this study, we provide several lines of evidence for a Ptr4CL-PtrHCT complex in *P. trichocarpa*, including (1) PtrHCT RNAi downregulated transgenic trees (Figure 2), (2) pair-wise combinations of BiFC (Figure 3), (3) protein pull-down from SDX extracts (Figure 4) and (4) enzyme activity using recombinant proteins (Figures 5, 6). By conducting the rule-based modeling and evolutionary computation, the most likely composition of the Ptr4CL and PtrHCT complex is predicted to have one subunit of Ptr4CL and two subunits of PtrHCTs (Figure 7). Finally, from chemical cross-linking of recombinant proteins and SDX crude extracts a protein band of ∼160 kDa was detected, which confirmed the predicted stoichiometry of a Ptr4CL-PtrHCT complex (Figure 8). In addition to the identification of protein-protein interactions between Ptr4CL and PtrHCT, the presence of PtrHCTs can facilitate the CoA ligation activity of Ptr4CLs for both 4-coumaric acid and caffeic acid (Figures 5, 6). This complex could play an important role in regulating the metabolic flux for CoA ligation in monolignol biosynthesis.

### Evidence of a 4CL-HCT Complex and Its Physiological Function

In Arabidopsis, the three isoforms of 4CL possess different substrate preferences and expression patterns. At4CL1 was proposed to be the best candidate for lignin formation (Ehlting et al., 1999). At the early branch point of monolignol biosynthesis in Arabidopsis, AtHCT was found partially associated with the ER and connected to the membrane-bound CYP73A5 (AtC4H) and CYP98A3 (AtC3′H) (Bassard et al., 2012). Moreover, AtC3′H was proposed to be the most effective in driving protein association with At4CL1 and AtHCT to the ER, while wound repair from mechanical injury could enhance the re-localization of these enzymes (Bassard et al., 2012). Neither TAP tagging nor blue native gel mobility could detect the association of At4CL1 and AtHCT in their native forms (Zhang and Liu, 2015); therefore, the physiological role of the 4CL-HCT association in Arabidopsis has not yet been established.

It has been suggested that a phenylpropanoid metabolon could alter the enzyme activity of different isoforms of monolignol biosynthetic enzymes (Jorgensen et al., 2005). In stem-differentiating xylem (SDX) of *P. trichocarpa*, two Ptr4CL isoforms (Ptr4CL3 and Ptr4CL5) and two PtrHCT isoforms (PtrHCT1 and PtrHCT6) were identified as xylem-specific and xylem-abundant enzymes that participate in monolignol biosynthesis (Shi et al., 2010). First, the reciprocal BiFC assays supported the protein-protein interaction between the Ptr4CLs and PtrHCTs (Figure 3). Then, we detected the direct protein–protein interactions between the isoforms of Ptr4CL and PtrHCT using pull-down assays (Figure 4). Consistently, the interaction between the isoforms of Ptr4CL and PtrHCT was shown to facilitate the enzyme activity of 4CL to promote CoA-ligation (Figures 5, 6).

Numerical analyses of the CoA ligation activity predicted a 1–2 Ptr4CL to PtrHCT protein ratio in the complexes (Figure 7). Besides the predominately predicted 1:2 ratio of Pt4CL-PtrHCT complex, there are other possible combinations predicted for the Ptr4CL5-PtrHCTs enzyme complex, such as a 1–3 ratio for Ptr4CL5-PtrHCT1 complex (Figures 7C,G) and a 1–1 ratio for Ptr4CL5-PtrHCT6 complex (Figure 7H), indicating that some specific interactions between Ptr4CL5 and PtrHCTs were able to be captured by our numerical analyses when different isoforms dominate in a well-controlled environment. However, a 1:2 ratio of Pt4CL-PtrHCT complex is experimentally confirmed by chemical cross-linking using SDX plant protein extracts (Figure 8).

An alternative role for the interaction between Ptr4CL and PtrHCT may be to protect 4-coumaroyl-CoA without it being released into the cytosolic pool, considering that the CoA thioesters, such as 4-coumaroyl-CoA, are relatively unstable metabolites and can be easily hydrolyzed in protein extracts (Loscher and Heide, 1994; Liang et al., 2010). The formation of a Ptr4CL-PtrHCT complex may facilitate the synthesis of CoA thioesters while further directing metabolic flux toward downstream monolignol biosynthesis without competing with other pathways. Moreover, in sorghum (*Sorghum bicolor*), a structural analysis of the SbHCT shows a significant affinity of SbHCT for 4-coumaroyl-CoA (1.6 μM) and a negligible affinity for shikimic acid, which suggests that 4-coumaroyl-CoA preconditions the binding of shikimic acid to SbHCT (Walker et al., 2013). Therefore, the Ptr4CL-PtrHCT complex could ensure the efficient binding of CoA thioesters to PtrHCT.

### Protein–Protein Interactions in Phenylpropanoid Metabolism

There are well-described mechanisms in the secondary metabolism of plants to effectively produce specific metabolites while avoiding metabolic interference, such as compartmentation, metabolic channeling, and metabolon formation (i.e., multienzyme complexes) (Ovádi, 1991; Jorgensen et al., 2005; Zhang and Fernie, 2020). These mechanisms can possess physiologically significant regulatory roles, such as relief of kinetic constraints, regulation of catalytic efficiency, sequestration of labile/toxic intermediates, and coordination of metabolic crosstalk to control the flux of metabolic sequences (Winkel-Shirley, 2001; Nakayama et al., 2019). These regulatory controls can facilitate the effective utilization of intermediates and modulate the carbon/energy flux among the branched pathways that often function concurrently in the same cell (Luckner, 1984; Weid et al., 2004; Winkel, 2004; Jorgensen et al., 2005; Ralston and Yu, 2006; Laursen et al., 2015; Bassard and Halkier, 2018). Metabolons are typically described as protein-protein interactions between ‘soluble’ enzymes that might be anchored to biological membranes or cytoskeletal elements, either by structural proteins or membrane-bound “anchor” proteins such as P450s. Early studies on protein-protein interactions suggested metabolite compartmentation or metabolic channeling in flavonoid biosynthesis (Fritsch and Grisebach, 1975; Jacques et al., 1977; Hrazdina et al., 1978; Stafford, 1981), isoflavonoids (Dixon et al., 1998; He and Dixon, 2000; Liu and Dixon, 2001; Waki et al., 2016), and monolignols (Czichi and Kindl, 1975, 1977; Hrazdina and Wagner, 1985b). For flavonoid biosynthesis, several members within specific metabolons were identified in different plant species, indicating the existence of the functional flavonoid metabolons (Dastmalchi et al., 2016; Fujino et al., 2018; Mameda et al., 2018; Nakayama et al., 2019).

In 1985, an ER-associated multienzyme complex for monolignol biosynthesis was proposed (Stafford, 1974b). Later, metabolic complexes for G and S monolignol biosynthesis were suggested, while COMT and CCoAOMT/CCR were thought to have independent metabolic routes (Dixon et al., 2001). Physical evidence for separate monolignol complexes is lacking, and the identification of lignin metabolons remains ongoing. Several protein complexes had been identified in monolignol biosynthesis, including a PAL-C4H complex (Rasmussen and Dixon, 1999; Achnine et al., 2004), a MSBP/P450 complex (Gou et al., 2018), a C3′H/C4H complex (Chen et al., 2011), a 4CL complex (Chen et al., 2014), and a CAD/CCR complex (Yan et al., 2019). In contrast to flavonoid biosyntheses, the regulatory effects of protein complexes in monolignol biosynthesis may support mechanisms other than metabolic channeling (Pettersson, 1991), such as feedback inhibition of PAL by cinnamate (Blount et al., 2000), the biochemical coupling of PAL-C4H (Ro and Douglas, 2004), and the absence of substrate channeling through a MSBP/P450 complex (Zhang and Fernie, 2020). In *P. trichocarpa*, the evidence of metabolic channeling remains debatable because exogenous metabolites can be used by the C3′H/C4H complex, and all 24 endogenous monolignol precursors can be detected in the crude SCW protein extract (Chen et al., 2011).

### Identification of a Lignin Metabolon

In flavonoid biosynthesis, the interactions among proposed metabolon enzymes vary between plant species, implying species-dependent metabolon formations for the diverse phenylpropanoids *in planta*, especially flavonoids (Nakayama et al., 2019). The flavonoid metabolon identified in snapdragon involved all FNSII, CHS, CHI, and DFR enzymes, while, in torenia, CHS was not found to interact with the proposed enzymes within the metabolon (Fujino et al., 2018). In hops, an active membrane-anchored metabolon for xanthohumol (prenylated flavonoid) biosynthesis has recently been identified. CHIL2, a non-catalytic CHI-like protein (CHIL), interacts with CHS_H1 (a hop specific CHS) and a membrane-bound prenyltransferase (PT1L) (Ban et al., 2018). Using Förster resonance energy transfer detected by fluorescence lifetime imaging microscopy (FLIM-FRET) in Arabidopsis protoplasts, CHS was identified to interact with flavonol synthase 1 (FLS1) or DFR to organize the flavonoid metabolon in a competitive manner to further modulate the metabolic flux into branching pathways (Crosby et al., 2011). Similarly, the identified enzyme complex of Ptr4CL-PtrHCT can be formed in a collaborative manner with the previously identified Ptr4CL3-Ptr4CL5 complex (Chen et al., 2014) or in a competitive mechanism in the lignin metabolon to modulate the CoA ligation flux for monolignol biosynthesis. The evidence of Ptr4CL-PtrHCT complex formation could be useful to investigate the possible role of these complexes or could be further integrated into a metabolic flux analysis for the identification of a lignin metabolon.

Moreover, crosstalk between metabolic enzymes among different metabolons should also be considered. In the CHI Arabidopsis flavonoid mutant (*tt5*), deficient for the major extractable leaf flavonols, the simultaneous downregulation of the sinapate ester biosynthesis was also observed even with the full capacity of the monolignol pathway (Li et al., 1993). For the production of isoflavonoids in soybean roots, GmCHR5, one of the 11 GmCHR paralogs, is the key enzyme for the physiological accumulation of 5-deoxyisoflavonoids by spatial-specific incorporation into the flavonoid metabolon (Mameda et al., 2018). Two other paralogs of GmCHR, GmCHR1, and GmCHR6, are proposed to involve the production of isoflavonoids for defense against microbial pathogens (Nakayama et al., 2019). Of the 17 Ptr4CL genes identified in the genome of *P. trichocarpa*, Ptr4CL1 and Ptr4CL17 also showed some specificity for differentiating xylem (Shi et al., 2010), despite their low transcript levels. These low abundance Ptr4CL paralogs could also participate in the lignin metabolon, which should be explored along with the Ptr4CL3 and Ptr4CL5. Moreover, the crosstalk between flavonoid and lignin metabolons is also possible by sharing a similar intermediate metabolite, 4-coumaroyl-CoA. Ptr4CL4 in *P. trichocarpa* (Shi et al., 2010) and 4CL2 in *P. tremuloides* (Hu et al., 1998) have both been associated with the biosynthesis of flavonoids and other soluble phenolics, which could be candidates for future investigation of protein–protein interactions.

The isolation of intact metabolons can be challenging if the protein-protein interactions in metabolons are weak or transient (Jorgensen et al., 2005; Nakayama et al., 2019). PAL-C4H is mediated by weak interactions that easily disassociate depending on the integrity of membranes during the purification (Hrazdina, 1992), treatment with ethylene (Czichi and Kindl, 1975), or under different physiological states (Luckner, 1984). In Arabidopsis, a loose association of P450s with HCT and 4-CL1 was observed (Bassard et al., 2012). Using an Y2H assay, At4CL1 had a physical interaction with AtC4H and AtC3′H, while AtCCR1 interacted with AtC4H (Gou et al., 2018). In *P. trichocarpa*, four monolignol protein complexes were identified, including PtrC3′H-PtrC4H, Ptr4CL3-Ptr4CL5, Ptr4CL-PtrHCT, and PtrCAD-PtrCCR. However, the complex formations for other monolignol biosynthetic enzymes, such as COMT, CCoAOMT, have not yet been linked to a lignin metabolon, and the relationships between the enzyme complex during the early steps of monolignol biosynthesis and the later steps such as F5H for downstream G- and S- monolignol biosynthesis remain unknown.

In the past, traditional techniques were conducted to illustrate the possible membrane-bound ER-resident protein complexes of phenylpropanoid metabolism, such as feeding labeled intermediates, cellular fractionation, co-immunolocalization, and gene co-expression analysis (Winkel-Shirley, 1999). Several successful techniques for studying protein-protein interactions could also be useful for future endeavors to investigate the lignin metabolon (Bassard and Halkier, 2018), such as two-hybrid assay (Ehlert et al., 2006), luciferase complementation imaging assay (LuCIA) (Ban et al., 2018), cross-linking/mass spectrometry (XL-MS) (Tang and Bruce, 2010), enzyme reconstitution (Mork-Jansson and Eichacker, 2019), microencapsulation (Anderson, 1974), and cryo-electron microscopy (cryo-EM) (Du et al., 2018). To reveal whether the existence of a cell-type-specific or a G/S-specific lignin metabolon is an intriguing topic for understanding the specific lignin deposition among different plant species. Metabolons can also vary greatly in physical stability, and the difference between an enzyme that presents in a defined stoichiometric ratio and a metabolon is neither precise nor absolute (Jorgensen et al., 2005). There are still many unanswered questions that need to be explored, including the possibility of an interactome among all the monolignol biosynthetic enzymes, regulatory effects on metabolic flux between phenylpropanoid metabolisms, dynamic compositions of the lignin metabolon for differential monolignol deposition, and utilization of the isoforms of monolignol biosynthetic enzymes in response to biotic and abiotic stresses.

## Conclusion

In the phenylpropanoid pathway, both flavonoid and monolignol biosyntheses are based on enzymes catalyzing sequential reactions. It is reasonable to investigate the spatial separation, competition, crosstalk, and affinities between the monolignol biosynthetic enzymes together with the intracellular distribution/transportation of the metabolites. These factors should play essential roles in modulating the functions of the monolignol enzyme complexes, leading to distinct regulatory and physiological roles in a lignin metabolon (Biała and Jasiński, 2018; Nakayama et al., 2019). Besides the previously identified monolignol complexes *in planta*, in this study, we provide direct evidence of a Ptr4CL-PtrHCT complex, which we propose to exist in *P. trichocarpa* for further modulating the CoA-ligation toward monolignol biosynthesis. The current knowledge is still far from sufficient to reconstruct a lignin metabolon. Efforts to unravel the supramolecular structure and function of this intriguing metabolon can shed some light on the enduring mystery of monolignol biosynthesis in different tissue types, differential lignin compositions, and the metabolic switches for specific metabolites *in planta*. A basic understanding of a monolignol metabolon could provide new strategies to manipulate the phenylpropanoid pathway to generate high-quality biomass for biofuel and other bioproducts.

## Materials and Methods

### Plant Materials

Clonal propagules of *Populus trichocarpa* (Nisqually-1) wildtype and the PtrHCT RNAi transgenic lines by Wang et al. (2018) were grown in a greenhouse in soil containing a 1:1 ratio of peat moss to potting-mix, using a 16 h light and 8 h dark photoperiod for 6 months before harvesting. The stem differentiating xylem (SDX) tissues were collected by scraping debarked stems using single-edge razor blades and instantly frozen in liquid nitrogen before storage at –80°C.

### Quantification of Transcripts and Proteins of Ptr4CLs and PtrHCTs in Stem-Differentiating Xylem of PtrHCT-RNAi Transgenic Lines and Wildtype

The quantitative data (transcript and protein abundances) of Ptr4CLs (Ptr4CL3 and Ptr4CL5) and PtrHCTs (PtrHCT1 and PtrHCT6) from the PtrHCT RNAi transgenic lines and the corresponding WT controls were retrieved from SDX transcriptomes (Wang et al., 2018) and absolute protein quantification from SDX crude protein extracts using protein cleavage isotope dilution mass spectrometry (PC-IDMS) analysis (Shuford et al., 2012). For WT or each line of transgenic plant, a biological replicate represents a pool of three to five clonally propagated trees.

### Enzyme Activity Assays of Ptr4CL in Stem-Differentiating Xylem Extracts

Stem-differentiating xylem crude protein extractions of WT and PtrHCT RNAi transgenic lines were conducted following our established protocols (Shi et al., 2010; Lin et al., 2015; Wang et al., 2018). Three grams of SDX was ground, extracted by ice-cold buffer (50 mM Tris-HCl, pH 7.5, 20 mM sodium ascorbate, 0.4 M sucrose, 100 mM NaCl, 5 mM DTT, 10% PVPP, 1 mM PMSF, 1 mg/mL pepstatin A and 1 mg/mL leupeptin), homogenized for 2 min on ice, and cleared twice by centrifugation at 4,000 × *g* for 15 min at 4°C. The supernatant was filtered, quantified the protein concentration of SDX extracts by the Bradford method, and stored at –80°C before enzyme assays.

Conditions for enzyme assays of Ptr4CL activities using SDX crude protein extracts also followed our established protocol (Liu et al., 2012). In brief, the activity assays of Ptr4CL reactions were examined using 20 μg SDX protein extracts at 37°C for 30 min with 50 μM substrate (final concentration) in the assay solution [50 mM Tris–HCl buffer (pH 7.5), 2.5 mM MgCl_2_, 5 mM ATP, 0.2 mM CoA] to a final volume of 100 μL. The reaction was terminated by the addition of 5 μL of 3 M trichloroacetic acid (TCA). Each reaction was repeated three times. After the mixture was centrifuged at 20,000 × *g* for 20 min, 90 μL of supernatant reaction mixture was transferred to an HPLC sample vial and 75 μL of supernatant was injected directly for HPLC analysis following (Liu et al., 2012).

### Purification of Recombinant Proteins and *in vitro* Enzyme Assays of Ptr4CLs

The procedures for expressing and purifying individual recombinant proteins (Ptr4CL3, Ptr4CL3, PtrHCT1, or PtrHCT6) and *in vitro* enzyme assays of Ptr4CLs were performed as previously described (Wang et al., 2014; Lin et al., 2015). In brief, a 100 μL reaction containing final concentration of 50 μM substrate (4-coumaric acid or caffeic acid), 0.2 mM CoA, 5 mM ATP and 2.5 mM MgCl_2_ in 50 mM Tris-HCl buffer (pH 8.0 for Ptr4CL3 and pH 7.0 for Ptr4CL5) starts with a final enzyme concentration of 10 nM of purified recombinant Ptr4CLs for a reaction time of 10 min at 40°C. The concentrations of recombinant PtrHCT in the enzyme assays were indicated in the corresponding figure legends.

### Bimolecular Fluorescence Complementation Assay

The coding sequences of PtrHCT6, PtrHCT1, Ptr4CL3, Ptr4CL5 without the stop codons were obtained by PCR using specific primer sets (Supplementary Table 1) and cloned using the pENTR^TM^ /D-TOPO^TM^ Cloning Kit (K240020, Invitrogen Carlsbad, CA, United States), and then recombined into the BiFC destination vector. All plasmids for bimolecular fluorescence complementation (BiFC) were prepared by CsCl gradient ultracentrifugation (Meselson et al., 1957; Radloff et al., 1967; Sambrook et al., 1982; Tartof, 1987; Sambrook and Russell, 2006). Each pair of the enzyme genes (Ptr4CL3-YFP^N^ + PtrHCT1-YFP^C^, Ptr4CL3-YFP^N^ + PtrHCT6-YFP^C^, Ptr4CL3-YFP^N^ + Ptr4CL3-YFP^C^, Ptr4CL3-YFP^N^ + Ptr4CL5-YFP^C^, Ptr4CL5-YFP^N^ + PtrHCT1-YFP^C^, Ptr4CL5-YFP^N^ + PtrHCT6-YFP^C^, Ptr4CL5-YFP^N^ + Ptr4CL3-YFP^C^, Ptr4CL5-YFP^N^ + Ptr4CL5-YFP^C^, PtrHCT1-YFP^N^ + PtrHCT1-YFP^C^, PtrHCT1-YFP^N^ + PtrHCT6-YFP^C^, PtrHCT1-YFP^N^ + Ptr4CL3-YFP^C^, PtrHCT1-YFP^N^ + Ptr4CL5-YFP^C^, PtrHCT6-YFP^N^ + PtrHCT1-YFP^C^, PtrHCT6-YFP^N^ + PtrHCT6-YFP^C^, PtrHCT6-YFP^N^ + Ptr4CL3-YFP^C^, PtrHCT6-YFP^N^ + Ptr4CL5-YFP^C^) were co-transfected into the SDX protoplasts. As negative controls (Ptr4CL3-YFP^N^ + Gus-YFP^C^, Ptr4CL5-YFP^N^ + Gus-YFP^C^, PtrHCT1-YFP^N^ + Gus-YFP^C^, PtrHCT6-YFP^N^ + Gus-YFP^C^) were co-transfected into the SDX protoplasts.

The transient expression of BiFC in SDX protoplasts followed our previously established protocol (Lin et al., 2014). After incubation for 12 ∼ 16 h, SDX protoplasts were collected and examined under a ZEISS LSM 700 fluorescence microscope. The excitation wavelength and the emission wavelength were 515 and 525 nm, respectively.

### Pull-Down Assays

Coding sequences of Ptr4CL3, Ptr4CL5, PtrHCT1, and PtrHCT6 without stop-codons were transferred from the pENTR gateway vector into *pET101-DEST* (C-terminal 6 × His tag) expression vector (K10101, Invitrogen) to generate the *pET101::*Gene-6 × His plasmids for recombinant protein expression in *Escherichia coli*. The procedures for the expression and purification of recombinant proteins were previously described (Wang et al., 2014; Lin et al., 2015). A pET*101:Gus-6* × *His* control was included with each pull-down to detect non-specific binding proteins (Liu et al., 2012; Wang et al., 2012; Chen et al., 2013). Recombinant 6 × His fused monolignol enzymes (60 μg) were individually incubated as bait protein in 50 mL of crude SDX protein extracts (0.4 mg/mL) at 4°C for 2 h. The protein mixtures were then affinity purified using immobilized nickel affinity chromatography to isolate interacting proteins bound to the recombinant monolignol bait protein. The affinity-purified proteins were analyzed by western blotting using polyclonal anti-Ptr4CL3, anti-Ptr4CL5, anti-PtrHCT1, and anti-PtrHCT6 antibodies as previously described (Chen et al., 2014).

### Mechanistic Modeling and Numerical Analysis

Mechanistic modeling was carried out by a previously developed rule-based algorithm and evolutionary computation algorithm for monolignol enzymes (Chen et al., 2014; Song, 2014). The algorithmic evolutionary processes automatically search and optimize the best solution for complex non-linear problems (Supplementary Figure 4). The method identifies possible components and interactions in multi-enzymatic reactions and suggests model structures and mathematical equations representing the mechanism of multi-enzymatic reactions.

The model is based on the experimental results of Ptr4CL CoA ligation reactions in mixtures of Ptr4CL and PtrHCT and evaluated by the numerical analysis of evolutionary computation. Two different numerical methods were used, the root mean squared error (RMSE) and Bayesian information criterion (BIC). The lower values of RMSE and BIC indicate the better models. The differences between the predicted values and experimental values are measured by RMSE. The lower values of RMSE and BIC indicate the better models. Adding on the goodness-of-fit of the model, BIC also reflects the model complexity using a penalty term for the number of parameters (Schwarz, 1978).

### Chemical Cross-Linking of Stem-Differentiating Xylem Proteins and Recombinant Proteins

Dithiobis (succinimidyl propionate) (DSP) (22585, Thermo Scientific, Waltham, MA, United States) was used as the cross-linker for the detection of protein-protein interactions. A 50 mM solution of DSP cross-linker was prepared by dissolving 10 mg of DSP in 495 μL of DMSO.

For cross-linking of SDX proteins, the SDX proteins were extracted using 25 mM sodium phosphate buffer (pH 7.4) instead of the 50 mM Tris-HCl buffer. The detailed cross-linking process of SDX proteins was previously described (Chen et al., 2011). For cross-linking of recombinant proteins, Ptr4CL3 or Ptr4CL5 was diluted in phosphate buffered saline (PBS) to a final concentration of 200 nM and mixed with individual aliquots of recombinant proteins of PtrHCT1 or PtrHCT6 (200 nM). DSP was then added to the protein mixtures at a 20-fold molar excess (20:1 DSP:protein). The protein cross-linking was carried out at room temperature for 5 min and quenched by the addition of 50 mM Tris-HCl (pH 7.5). The DSP-treated and the untreated protein samples were then incubated with sample loading buffer [2% SDS, 200 mM Tris-HCl (pH 6.8), 40% glycerol, and 0.08% bromophenol blue] and heated to 95°C for 5 min, separated by SDS-PAGE, and analyzed by western blotting using Ptr4CL3 and PtrHCT1 antibodies.

## Data Availability Statement

The datasets presented in this study can be found in online repositories. The names of the repository/repositories and accession number(s) can be found in the article/Supplementary Material.

## Author Contributions

C-YL and YS performed the enzyme activity analyses. RS and CY generated the PtrHCT-RNAi transgenic poplar. JL performed the enzyme activities of SDX extracts. ST-A and PL performed the RNAseq analysis and protein quantification. JS performed the mechanistic modeling and numerical analysis. C-YL, YS, and H-CC performed the pull-down and cross-linking experiments. C-YL, YS, JS, CW, DM, Y-CL, RRS, JW and VC analyzed the data. C-YL, YS, JS, and JW wrote the manuscript. JW, RRS, and VC edited the manuscript. C-YL, YS, and JS contributed equally to this work. All the authors contributed to the article and approved the submitted version.

## Conflict of Interest

The authors declare that the research was conducted in the absence of any commercial or financial relationships that could be construed as a potential conflict of interest.

## Publisher’s Note

All claims expressed in this article are solely those of the authors and do not necessarily represent those of their affiliated organizations, or those of the publisher, the editors and the reviewers. Any product that may be evaluated in this article, or claim that may be made by its manufacturer, is not guaranteed or endorsed by the publisher.

## Abbreviations

AC: affinity chromatography
BiFC: biomolecular fluores-cence complementation
DSP: dithiobis (succinimidyl propionate)
IP: immuno-precipitation
LCC: lignin–carbohydrate complexes
PC-IDMS: protein cleavage isotope dilution mass spectrometry
RNAi: RNA interference
RPM: reads per million
SCW: secondary cell wall
SDX: stem-differentiating xylem
TAP: tandem affinity purification (TAP)
Y2H: yeast two-hybrid.
