# Supplementary material for: Enzyme Complexes of Ptr4CL and PtrHCT Modulate Co-enzyme A Ligation of Hydroxycinnamic Acids for Monolignol Biosynthesis in Populus trichocarpa
Source: Front Plant Sci. 2021 Oct 6;12:727932. doi: 10.3389/fpls.2021.727932 (PMC8527181; doi:10.3389/fpls.2021.727932)

**Manuscript title:**

Enzyme Complexes of Ptr4CL-PtrHCT Modulate Co-enzyme A Ligation of Hydroxycinnamic Acids for Monolignol Biosynthesis in *Populus trichocarpa*

**Additional file**

File type: PDF

**Contents:**

- **Supplemental Table S1.** List of primer set for construction of BiFC plasmids.
- **Supplemental Table S2.** The optimized models of the reaction rates for Ptr4CLs towards 4-coumaric acid (Reaction A) in protein complex with PtrHCTs.
- **Supplemental Table S3.** The optimized models of the reaction rates for Ptr4CLs towards caffeic acid (Reaction B) in protein complex with PtrHCTs.
- **Supplemental Figure S1.** Transcript abundance of SDX-specific PtrHCTs in WT, PtrHCT1 or PtrHCT6 RNAi-downregulated transgenic lines.
- **Supplemental Figure S2.** Protein abundance of SDX-specific Ptr4CLs and PtrHCTs in WT, PtrHCT1 or PtrHCT6 RNAi-downregulated transgenic lines.
- **Supplemental Figure S3.** Antibodies specificities for PtrHCTs and Ptr4CLs confirmed by western blots using SDX extracts.
- **Supplemental Figure S4.** Rule-based modeling and evolutionary computation for multi-enzymatic reaction modeling framework.
- **Supplemental Figure S5.** The goodness-of-fit of the optimized model with experimental data under the respective different conditions
- **Supplemental Figure S6.** The full and uncropped western blots for Figures 4 and 8.

**Supplemental Table S1.** List of primer set for construction of BiFC plasmids.

| **Primer** | **Sequence** | **Accession No.** | **Amplicon** |
| --- | --- | --- | --- |
| Ptr4CL3-F | *CACC*ATGGACGCCATAATGAATTCACAA | EU603298.1 | 1624 bp |
| Ptr4CL3-R | TATGCCTGGCAACGTTTCTCTCAG |  |  |
| Ptr4CL5-F | *CACC*ATGGATACAATAACAAAGCAAAAA | EU603299.1 | 1633 bp |
| Ptr4CL5-R | CTTTTGCAAACCACCTGCCAACCT |  |  |
| PtrHCT1-F | *CACC*ATGATAATCAATGTGAAGGA | EU603313.1 | 1303 bp |
| PtrHCT1-R | TTCTTTAATGTCATATATAAACTTCT |  |  |
| PtrHCT6-F | *CACC*ATGATAATCAACGTGAAGGAGTCA | EU603314.1 | 1297 bp |
| PtrHCT6-R | AATGTCATATATGAACTTCTCAAA |  |  |
| Gus-F | *CACC*ATGTTACGTCCTGTAGAAAC | S69414.1 | 1813 bp |
| Gus-R | TTGTTTGCCTCCCTGCTGCG |  |  |

^*^Italic bases are necessary for directional cloning into pENTR^TM^ vector.

^*^Underlined bases are the start codon of the gene.

**Supplemental Table S2.** The optimized models of the reaction rates for Ptr4CLs towards 4-coumaric acid (**Reaction A**) in protein complex with PtrHCTs.

A B C D

$C_{1}=\frac{k_{cat1}\cdot[S]}{K_{M1}+[S]}$ and $C_{3}=k_{cat1}\cdot[S]$ where $[S]$ is the concentration of 4-coumaric Acid, and $k_{cat1}$ and $K_{M1}$ are enzyme (A and B : 4CL3, C and D : 4CL5) kinetic parameters for 4-coumaric Acid reaction. $[E1t]$ is initial enzyme (A and B: 4CL3, C and D: 4CL5) concentrations. $[E_{C1}]$ represents the maximum value of the enzyme (A and C: HCT1, B and D: HCT6) that can be involved in the enzyme complex. $[E_{Cm}]$ represents the maximum concentration that the enzyme complex can have.

**Supplemental Table S3.** The optimized models of the reaction rates for Ptr4CLs towards caffeic acid (**Reaction B**) in protein complex with PtrHCTs.

A B C D

$C_{1}=\frac{k_{cat1}\cdot[S]}{K_{M1}+[S]}$ and $C_{3}=k_{cat1}\cdot[S]$ where $[S]$ is the concentration of caffeic acid, and $k_{cat1}$ and $K_{M1}$ are enzyme (A and B : 4CL3, C and D : 4CL5) kinetic parameters for caffeic acid reaction. $[E1t]$ is initial enzyme (A and B: 4CL3, C and D: 4CL5) concentrations. $[E_{C1}]$ represents the maximum value of the enzyme (A and C: HCT1, B and D: HCT6) that can be involved in the enzyme complex. $[E_{Cm}]$ represents the maximum concentration that the enzyme complex can have.

**
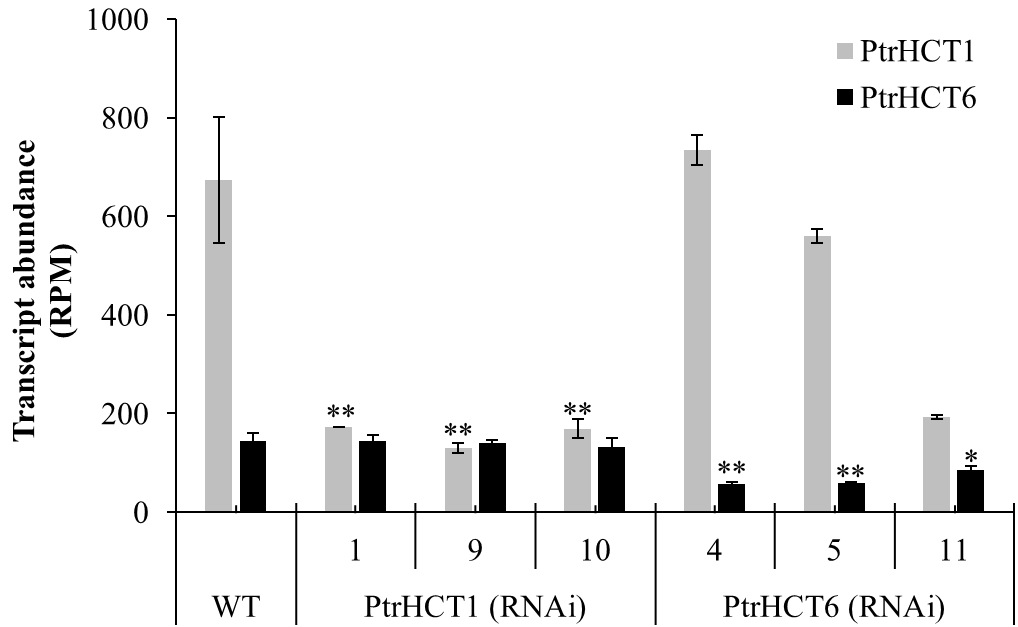
**

**Supplemental Figure S1.** Transcript abundance of SDX-specific PtrHCTs in WT, PtrHCT1 or PtrHCT6 RNAi-downregulated transgenic lines. Error bars represent SE of three replicates. RPM, reads per million. Statistical testing was performed using Student’s t-test (*, *p* < 0.05; **, *p* < 0.01).

**
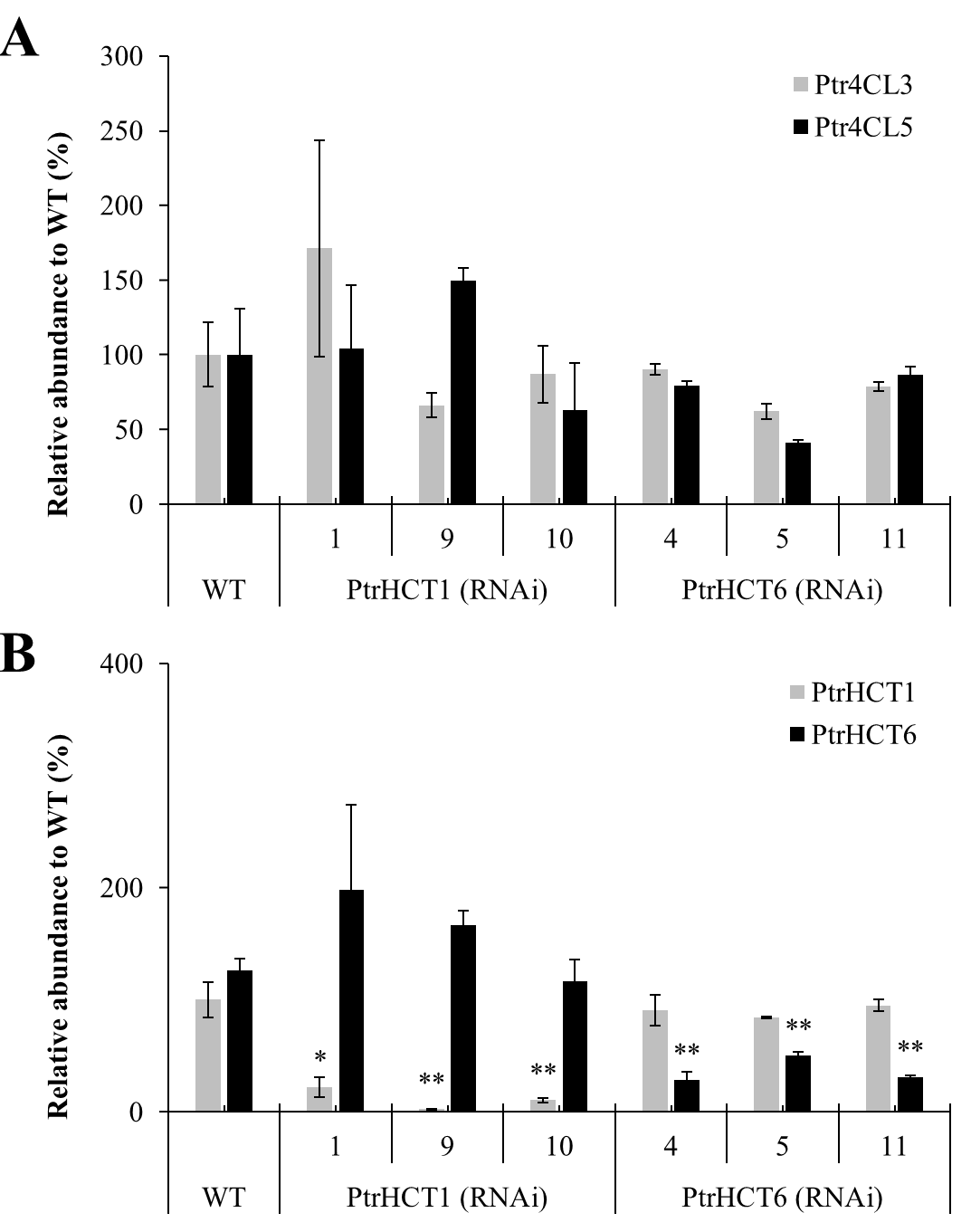
**

**Supplemental Figure S2.** Protein abundance of SDX-specific Ptr4CLs and PtrHCTs in WT, PtrHCT1 or PtrHCT6 RNAi-downregulated transgenic lines. Error bars represent SE of three replicates. Statistical testing was performed using Student’s t-test (*, *p* < 0.05; **, *p* < 0.01).


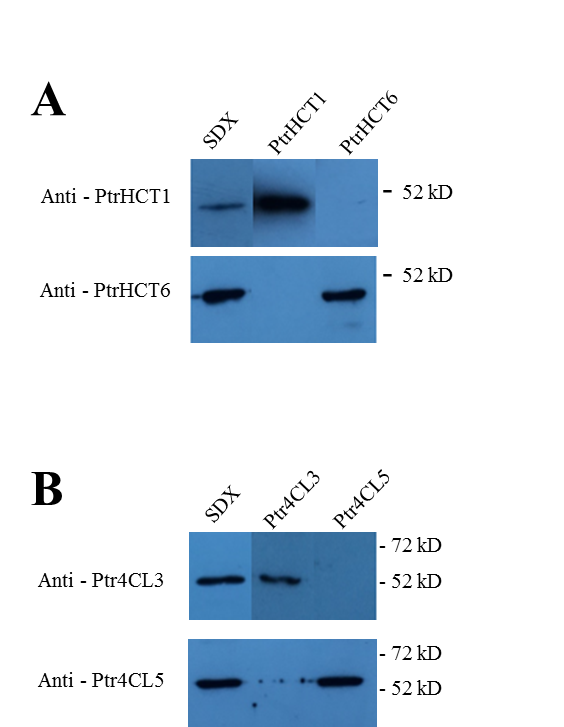


**Supplemental Figure S3.** Antibodies specificities for PtrHCTs and Ptr4CLs are confirmed by western blots using SDX extracts. **(A)** Specific detection of PtrHCT1 or PtrHCT6 using antibodies for PtrHCTs. **(B)** Specific detection of Ptr4CL3 or Ptr4CL5 using antibodies for Ptr4CLs.

**Supplemental Figure S4.** Rule-based modeling and evolutionary computation for multi-enzymatic reaction modeling framework. (Jina Song, Ph.D. Dissertation (2014)).


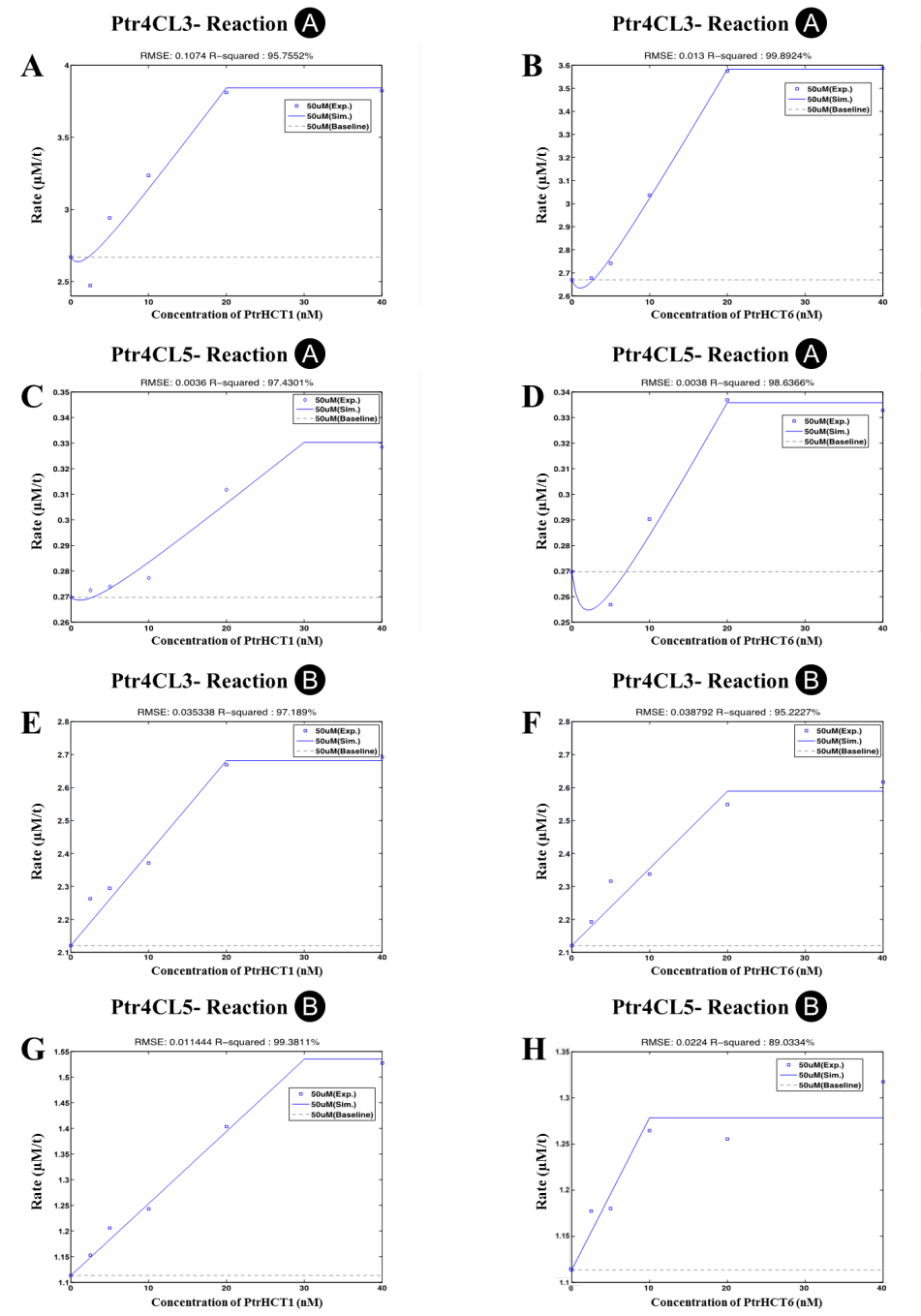


**Supplemental Figure S5.** The goodness-of-fit of the optimized model with experimental data under the respective different conditions. (**A**) Simulation Result of Reaction A for Ptr4CL3 with varied PtrHCT1 concentration. (**B**) Simulation Result of Reaction A for Ptr4CL3 with varied PtrHCT6 concentration. (**C**) Simulation Result of Reaction A for Ptr4CL5 with varied PtrHCT1 concentration. (**D**) Simulation Result of Reaction A for Ptr4CL5 with varied PtrHCT6 concentration. (**E**) Simulation Result of Reaction B for Ptr4CL3 with varied PtrHCT1 concentration. (**F**) Simulation Result of Reaction B for Ptr4CL3 with varied PtrHCT6 concentration. (**G**) Simulation Result of Reaction B for Ptr4CL5 with varied PtrHCT1 concentration. (**H**) Simulation Result of Reaction B for Ptr4CL5 with varied PtrHCT6 concentration. “X-axis” represents the enzyme concentration, “Y-axis” represents the reaction rate, and the lines have the fixed concentrations of substrates of 50μM. RMSE and R^2^ evaluate the goodness-of-fit numerically.

**Supplemental Figure S6.** The full and uncropped western blots for Figures 4 and 8.


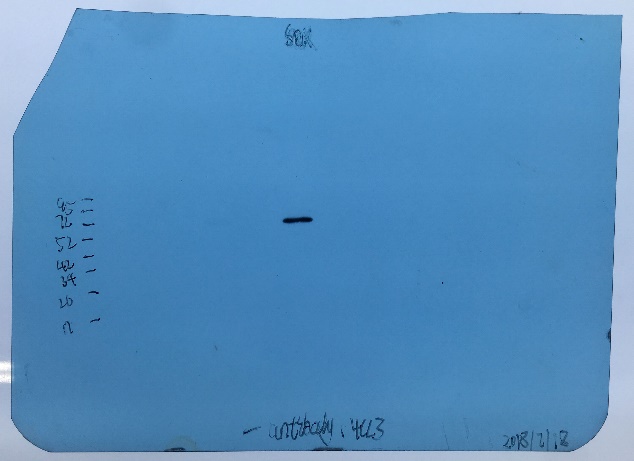


**Figure 4A** - SDX


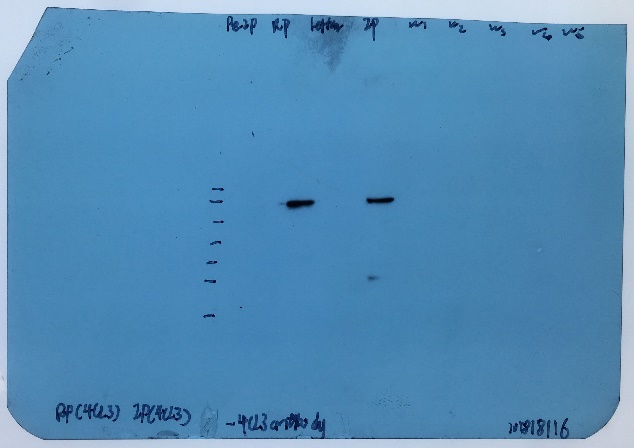


**Figure 4A** – Ptr4CL3


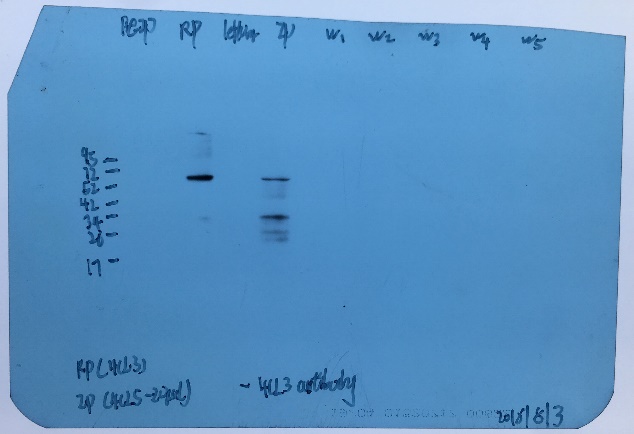


**Figure 4A** – Ptr4CL5


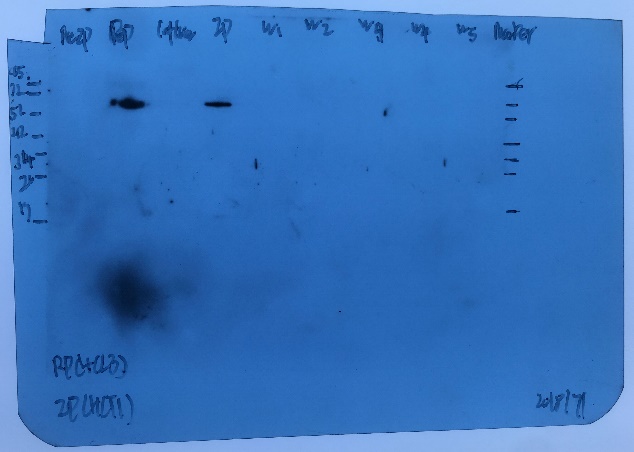


**Figure 4A** – PtrHCT1


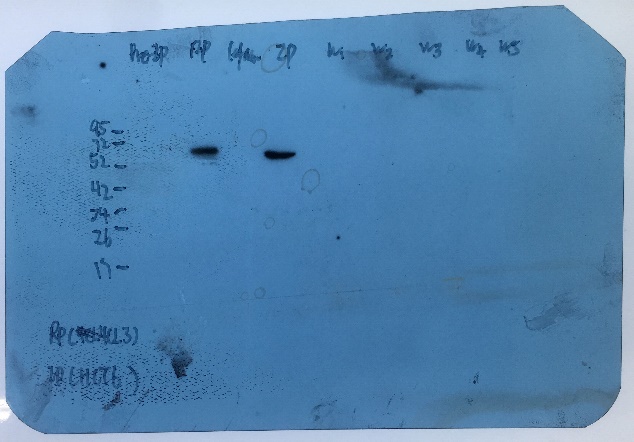


**Figure 4A** – PtrHCT6


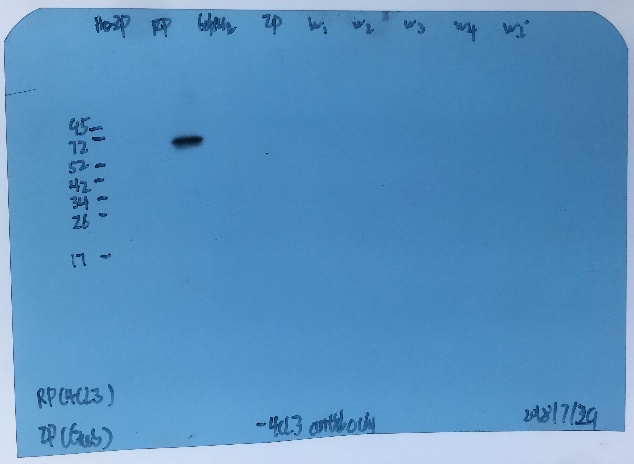


**Figure 4A** – GUS


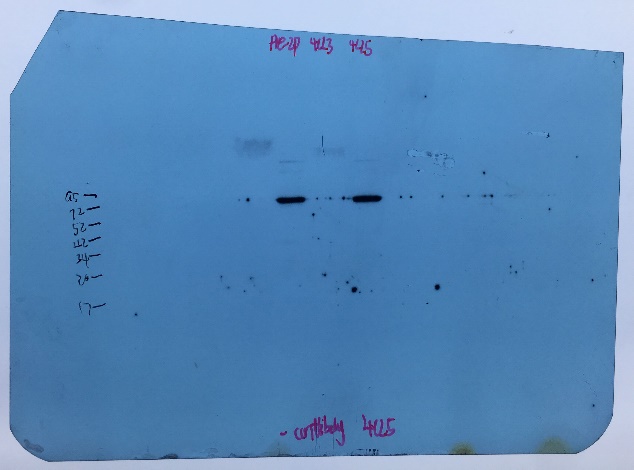


**Figure 4B** – SDX


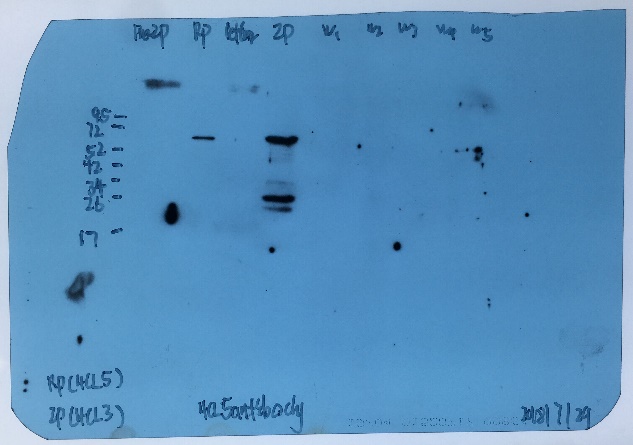


**Figure 4B** – Ptr4CL3


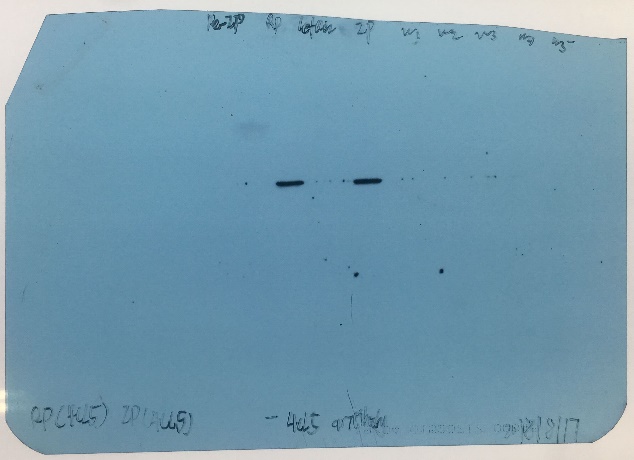


**Figure 4B** – Ptr4CL5


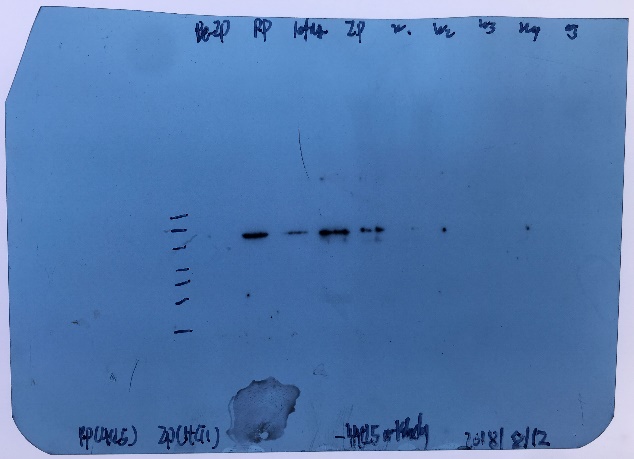


**Figure 4B** – PtrHCT1


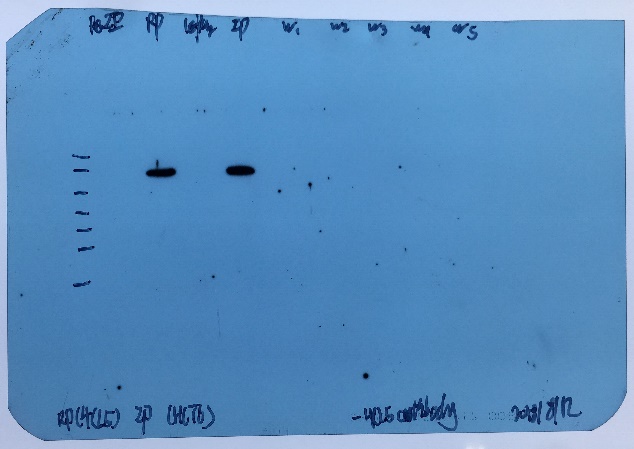


**Figure 4B** – PtrHCT6


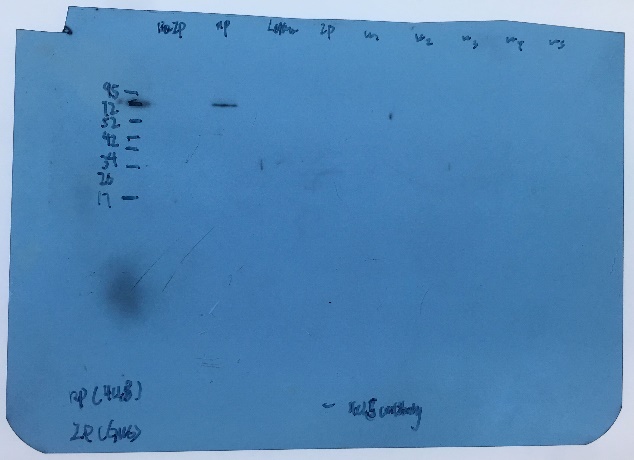


**Figure 4B** – GUS


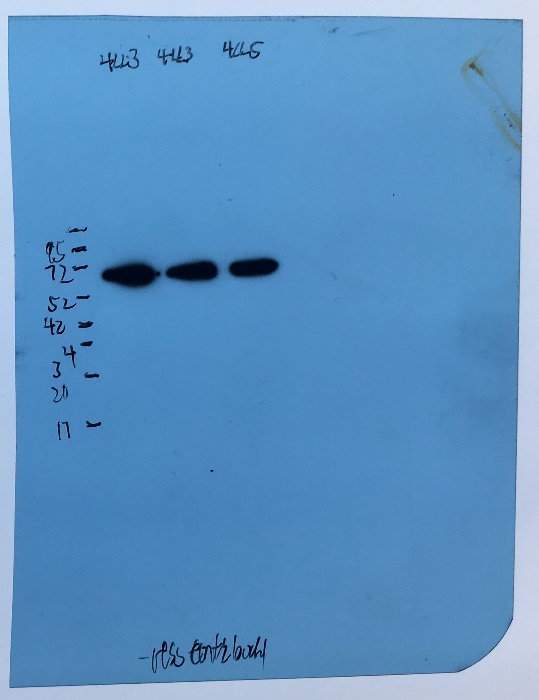


**Figure 4C** – Ptr4CL3 and Ptr4CL5


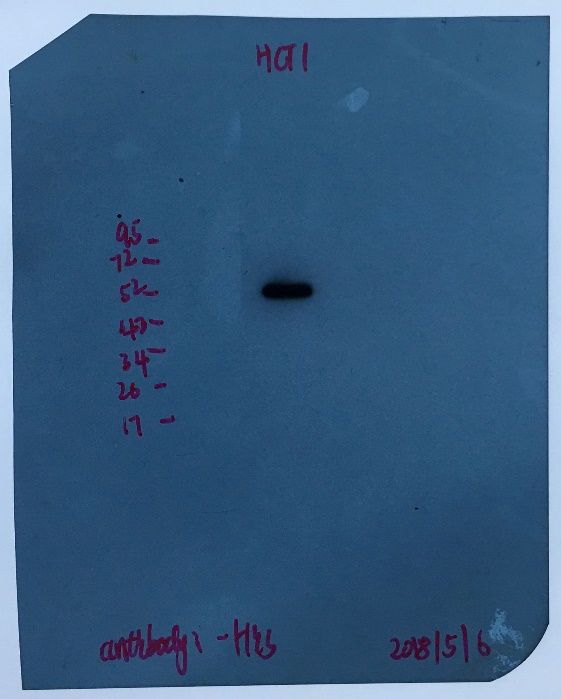


**Figure 4C** – PtrHCT1


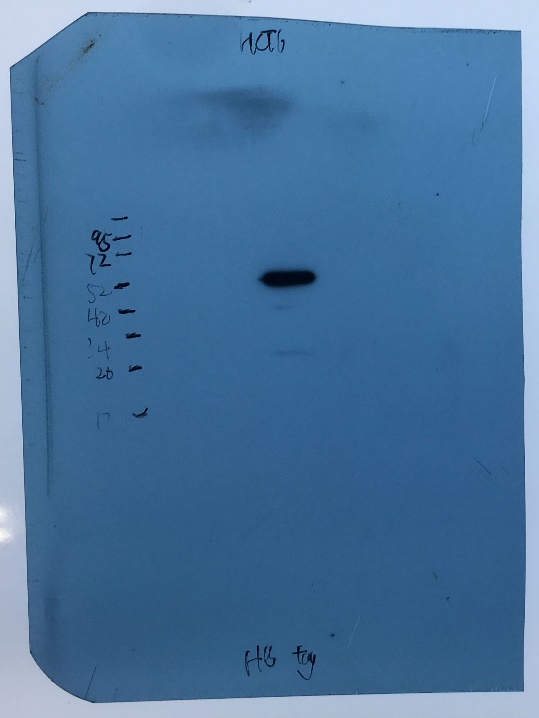


**Figure 4C** – PtrHCT6


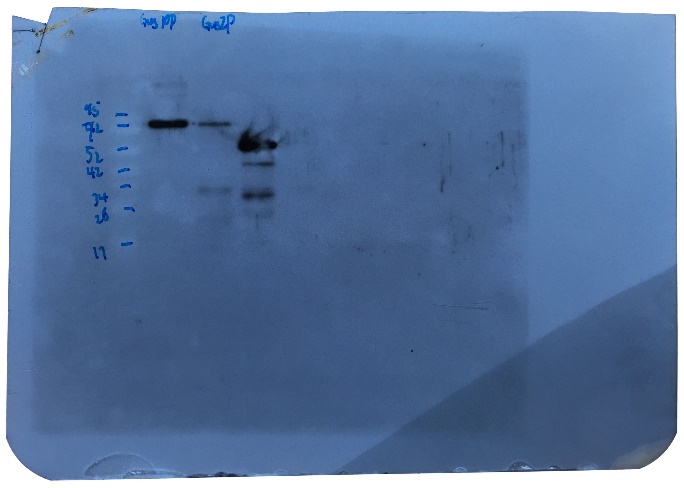


**Figure 4C** – GUS


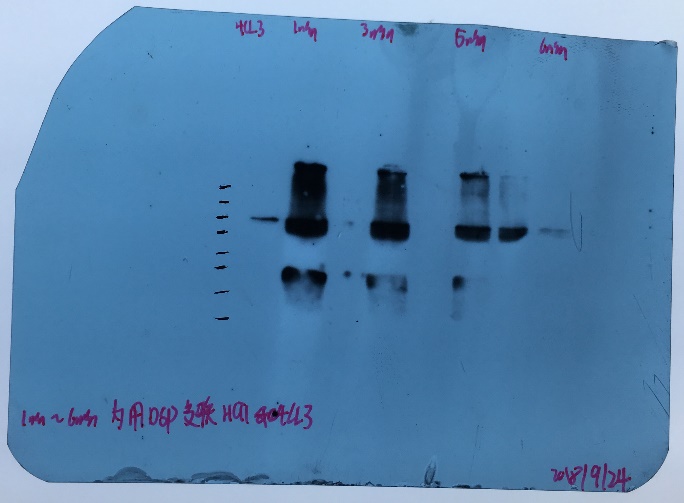


**Figure 8A** – Lanes 1 and 2


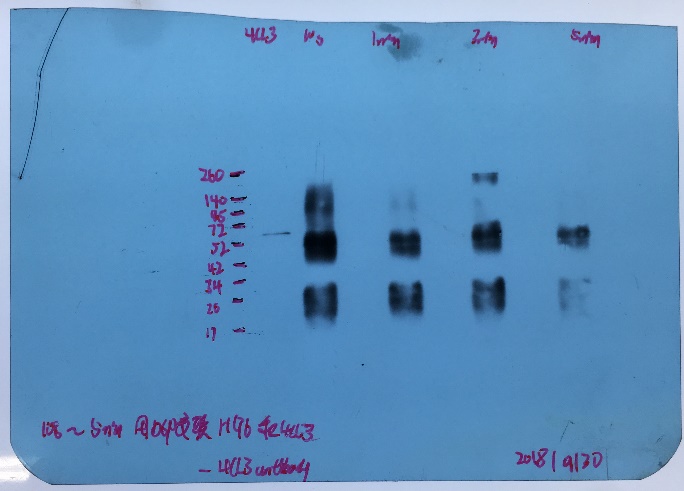


**Figure 8A** – Lanes 3 and 4

**Figure 8B** – Lanes 1 and 2


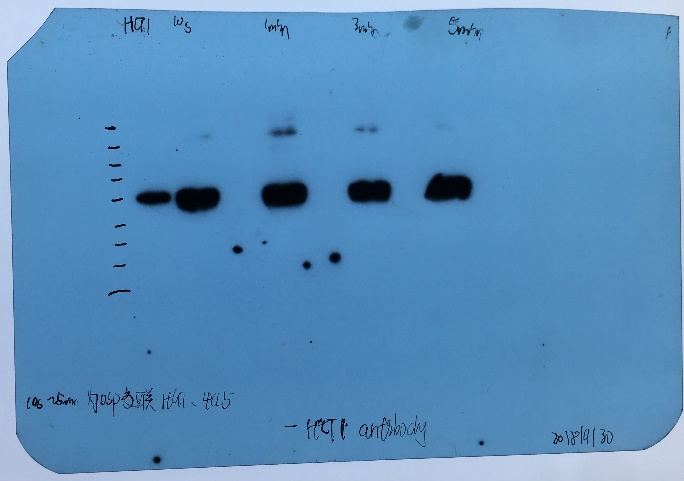


**Figure 8B** – Lanes 3 and 4


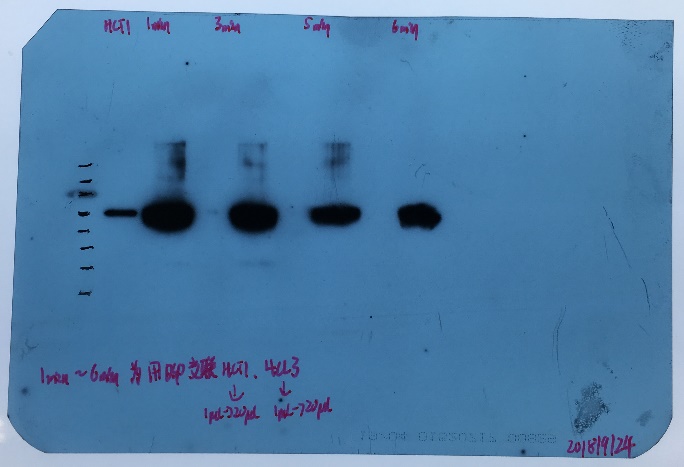


**Figure 8C**

**Figure 8D**


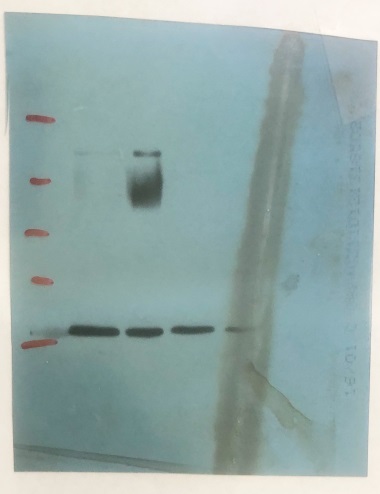

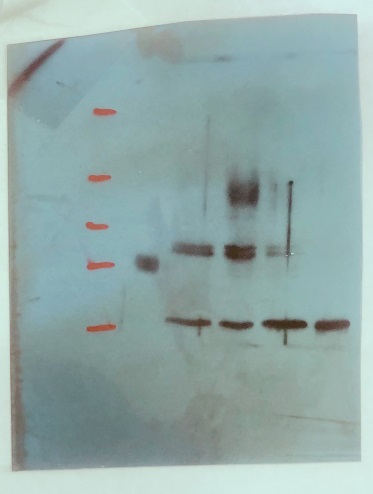

Supplement: Supplementary file 1 [file Data_Sheet_1.DOCX]
